# Medicine–Food Homology Bioactives for Polyendocrine Metabolic Ovarian Syndrome: Multi-Target Mechanisms and Functional Food Translation

**DOI:** 10.3390/molecules31203633

**Published:** 2026-10-12

**Authors:** Xiaoman Lv, Wenjuan Long, Zhongyang Hu, Mingqin Shi, Xinyao Li, Yujun Feng, Jiarong Du, Zhicheng Yang, Fang Peng, Liwei Xing, Dongdong Qin

**Affiliations:** 1 College of Pharmaceutical Science, Dali University, Dali 671003, China; lxm.cc@foxmail.com (X.L.); pengfang6556@aliyun.com (F.P.); 2 Key Laboratory of Traditional Chinese Medicine for Prevention and Treatment of Neuropsychiatric Diseases, Yunnan University of Chinese Medicine, Kunming 650500, China; likq20201029@163.com (W.L.); xh18286279638@163.com (Z.H.); fyyyj0727@163.com (Y.F.); xxr95822020@163.com (J.D.); 3 The First Clinical Medical College, Yunnan University of Chinese Medicine, Kunming 650500, China; shimingqin1998@163.com (M.S.); lxy9081720@163.com (X.L.); 4 Yunnan Academy of Agricultural Engineering, Kunming 650215, China; 18213014020@163.com

**Keywords:** polyendocrine metabolic ovarian syndrome, polycystic ovary syndrome, polyphenols, network pharmacology, PI3K/AKT signaling, gut microbiota, nanoencapsulation, bioavailability

## Abstract

Polyendocrine Metabolic Ovarian Syndrome (PMOS) is characterized by insulin resistance, hyperandrogenism, chronic inflammation, and oxidative stress, with a global prevalence of 10–13% and limited current drug treatments. Medicine–Food Homology (MFH) materials are rich in bioactive compounds such as polyphenols, flavonoids, and polysaccharides, offering potential multi-target nutritional interventions for PMOS, although safety data remain limited mainly to single compounds. A systematic search of the literature was conducted across PubMed/MEDLINE, Web of Science CNKI, and Wanfang Data, spanning from database inception to August 2026, utilizing predefined search terms and inclusion/exclusion criteria. This review summarizes research on MFH-derived bioactive compounds in PMOS, covering active component identification, network pharmacology-based target prediction, key signaling pathways (PI3K/AKT, AMPK, NF-κB, Nrf2/HO-1), and formulation strategies including green extraction, nano-encapsulation, and controlled release. It further discusses functional food development from five perspectives: meal replacement powders, functional teas, fermented beverages, dietary supplements, and compound formulations. Key bottlenecks hindering commercialization are analyzed, including insufficient high-quality clinical evidence, unclear dose–response relationships, and limited long-term stability data. Future directions are proposed, such as organoids, organ-on-a-chip, AI-driven formulation optimization, and international multicenter trials. In summary, MFH bioactive compounds show potential as multi-target interventions for PMOS; however, most mechanistic evidence comes from non-PMOS metabolic models, and direct PMOS-specific evidence is limited. Their translation from empirical use to evidence-based intervention requires PMOS-specific preclinical validation, randomized controlled trials, safety assessments, bioavailability evaluation, and standardized product validation, alongside collaborative efforts across research paradigms, technology development, and industrial translation.

## 1. Introduction

According to recent international consensus, polycystic ovary syndrome (PCOS) has been officially renamed polyendocrine metabolic ovarian syndrome (PMOS) to more accurately reflect its nature as a complex systemic disease affecting the endocrine, metabolic, and reproductive systems [1]. This nomenclature change was established through a multistep global consensus process published in *The Lancet*, involving 56 leading organizations and over 14,000 participants, including patients and health professionals across all world regions [1]. The consensus concluded that the term PCOS is inaccurate, as it implies pathological ovarian cysts while obscuring the disorder’s endocrine and metabolic features, contributing to delayed diagnosis and stigma. Given that most evidence synthesized herein was originally published under the term PCOS, literature searches used both “PCOS” and “PMOS” without date restriction, with PMOS used consistently throughout. PMOS is the most common endocrine disorder among women of childbearing age worldwide, with a prevalence of approximately 10–13%. Its clinical features include hyperandrogenism (hirsutism, hair loss, acne), metabolic disorders (insulin resistance, type 2 diabetes, increased cardiovascular risk), and reproductive dysfunction, and it significantly increases the risk of mental health disorders and endometrial cancer [2].

The pathophysiological mechanisms of PMOS are extremely complex, involving multiple interacting dysregulations across the neuroendocrine, metabolic, inflammatory, and reproductive systems [3]. Insulin resistance (IR) is the most critical metabolic abnormality in PMOS; approximately 65–70% of patients exhibit IR, which occurs independently of obesity. Hyperinsulinemia drives hyperandrogenism by enhancing ovarian sensitivity to luteinizing hormone (LH), inhibiting hepatic sex hormone-binding globulin (SHBG) synthesis, and disrupting the synergistic signaling pathway involving insulin-like growth factor (IGF) and follicle-stimulating hormone (FSH); in turn, hyperandrogenism exacerbates IR by promoting visceral fat accumulation, creating a vicious cycle [4]. A state of chronic low-grade inflammation is characterized by elevated levels of pro-inflammatory factors such as C-reactive protein (CRP), TNF-α, and IL-6, and exists independently of obesity; inflammatory factors exacerbate IR by inhibiting IRS-1/PI3K/AKT phosphorylation, while simultaneously activating the local ovarian inflammatory microenvironment and impairing granulosa cell function [5]. Regarding oxidative stress (OS), hyperandrogenism and IR synergistically induce excessive production of reactive oxygen species, and the imbalance in the antioxidant defense system further exacerbates impaired follicular development [6,7]. Gut microbiota dysbiosis contributes to the development of IR, hyperandrogenism, and metabolic disorders by mediating abnormalities in bile acid metabolism, altering short-chain fatty acid levels, and activating chronic inflammation [8]. Disruption of the Hypothalamic–Pituitary–Ovarian (HPO) axis manifests as an elevated LH/FSH ratio resulting from an increased frequency of gonadotropin-releasing hormone (GnRH) pulses; approximately 75% of patients with PMOS exhibit elevated LH levels. Excessive LH stimulates androgen synthesis in theca cells, while relative FSH deficiency inhibits aromatase activity in the granulosa cells, creating a vicious cycle of hyperandrogenism and arrested follicular development [9].

The current clinical management of PMOS primarily focuses on lifestyle interventions as a core strategy, encompassing dietary adjustments, physical exercise, and behavioral and psychological interventions, with the aim of improving metabolic, endocrine, and reproductive outcomes and providing a foundation for personalized treatment [10]. Current evidence suggests that when patients with PMOS achieve weight loss of more than 5% of their baseline weight through lifestyle interventions, IR is significantly improved, thereby reducing the stimulatory effect of hyperinsulinemia on the ovaries and restoring hormonal balance and ovulatory function [11]. However, lifestyle interventions alone face challenges in clinical practice, such as poor patient adherence and significant individual variability in response. Regarding pharmacological treatment, while oral contraceptives, insulin sensitizers (such as metformin), and antiandrogens can alleviate symptoms to some extent, they are associated with issues such as an increased risk of thrombosis, gastrointestinal adverse reactions, and concerns regarding long-term safety [12,13]. Due to the complex etiology and high phenotypic heterogeneity of PMOS, there is currently no universally applicable standardized treatment protocol.

In the search for safe and sustainable complementary treatment strategies, the concept of “Medicine–Food Homology” (MFH) has garnered increasing attention. This concept is rooted in the principle of “medicine and food share the same origin” in traditional Chinese medicine—that is, the same substance can serve as both food to provide nutrition and as medicine to exert therapeutic effects [14]. This concept aligns perfectly with the maxim of the ancient Greek physician Hippocrates: “Let food be your medicine, and medicine be your food.” To provide a clear regulatory context, the official definition and inclusion criteria of MFH substances are summarized below. According to the Regulations on the Management of the Catalog of Substances That Are Both Food and Traditional Chinese Medicinal Materials (National Health Commission Food Safety Document [2021] No. 36), substances designated as both food and traditional Chinese medicinal materials are defined as “substances traditionally consumed as food and listed in the Pharmacopoeia of the People’s Republic of China” [15]. The Regulations on the Management of the Catalog of Substances Traditionally Used as Both Food and Chinese Medicinal Materials, issued by the National Health Commission, stipulate four concurrent criteria for inclusion: a tradition of consumption as food; listing in the Chinese Pharmacopoeia; safety assessments showing no food-safety issues; and compliance with relevant laws and regulations, including those related to the protection of Chinese medicinal material resources [16]. Since its initial release in 2002, the catalog has been expanded through multiple announcements in 2019, 2023, and 2024, and by 2025 it comprises 106 substances. It should be noted that the “food-as-medicine” designation is jurisdiction-specific and plant-part-specific; the fact that a substance is listed as “food-as-medicine” in mainland China does not imply approval as a food ingredient under other regulatory regimes, and inclusion in the catalog typically applies only to specific plant parts [17]. Modern research has confirmed that MFH is rich in bioactive components, including polyphenols, polysaccharides, and dietary fiber, and has attracted considerable attention for its roles in modulating glucose and lipid metabolism, OS, and chronic inflammation; however, its effects in PMOS still require validation in PMOS-specific models and clinical trials [18]. The value of dietary bioactive compounds in the prevention and treatment of metabolic diseases has been widely recognized, laying a theoretical and practical foundation for precision nutritional interventions [19]. Nevertheless, existing evidence is largely limited to single components or crude extracts, and the core molecular basis underlying metabolic improvements by MFH, the chemical nature of its bioactive components, and their target sites within the PMOS pathological network remain insufficiently elucidated, hindering the transition of MFH strategies from empirical application to precision intervention. Although existing reviews on polyphenols or flavonoids in PCOS have explored their interventional potential from different perspectives, an integrated framework centered on PMOS that spans from the discovery of MFH active compounds to clinical translation has not yet been established. Balieiro et al. [20] demonstrated through a meta-analysis of RCTs that polyphenol supplementation reduced insulin, BMI, and LH levels in patients with PCOS, but focused solely on clinical phenotypes. Lv et al. [14] reviewed the concept of MFH, the classification of 102 listed substances, and their pharmacological mechanisms, but focused on general metabolic disorders and preclinical evidence. Wang et al. [21] summarized dietary sources, bioavailability enhancement, and functional food strategies for PCOS, but were restricted to flavonoids. Chen et al. [22] used network pharmacology to reveal that luteolin/quercetin regulate the MAPK axis via AKT1, EGFR, and INSR, but relied mainly on computational predictions and retained the PCOS nomenclature. None of these studies covered the full range of MFH categories. In contrast, the present review uses PMOS as a new nomenclature framework to construct a PMOS-specific full-chain analytical framework.

Bioactive compounds in food refer to biologically active components that occur naturally in food and, in addition to providing basic nutrition, can regulate physiological functions when consumed in appropriate amounts. These substances are widely distributed in plant-based foods and can be classified according to their chemical structure into polyphenols (flavonoids, phenolic acids, lignans, etc.), terpenoids, alkaloids, polysaccharides, and sulfur-containing compounds [23,24]. Their core characteristic lies in the fact that, when ingested as part of the diet, they exert biological effects by regulating redox balance, inflammatory signaling pathways, and hormonal metabolic networks [25,26]. Food bioactive compounds may offer multi-target regulatory potential and could serve as a potential bridge linking nutritional science and PMOS management.

Given the multidimensional nature of the pathophysiological mechanisms underlying PMOS and the limitations of existing interventions, bioactive compounds derived from MFH ingredients may offer a potential adjunctive strategy for PMOS due to their potential multi-target regulatory effects; however, their long-term safety, clinical efficacy, bioavailability, and product feasibility remain to be established. Furthermore, current research has largely focused on phenotypic observations of individual components, lacking a systematic understanding of their active substance basis, action targets, and molecular networks; moreover, the pathway from basic research to the development of functional food products has not yet been systematically mapped out. Unlike previous reviews that have largely catalogued individual bioactivities or single product categories, this review adopts an integrated, translation-oriented framework that connects active compound identification, molecular target prediction, formulation strategies, functional food development, and clinical translation bottlenecks. This review aims to summarize the full-chain research progress on bioactive compounds from MFH-derived foods in the management of PMOS, covering the following four aspects: (1) chemical classification and characterization methods of active components (systematic screening based on metabolomics and network pharmacology); (2) key molecular mechanisms for improving IR, regulating ovarian steroid synthesis, alleviating inflammation and OS, and modulating the HPO axis; (3) the application of pharmaceutical strategies, such as green extraction technologies, nano-encapsulation, and targeted controlled-release systems, to overcome application bottlenecks for active ingredients; (4) the development logic and principles of compound formulation design for product forms such as meal replacement powders, functional tea beverages, fermented beverages, and dietary supplements. Building on this foundation, this paper further analyzes the key bottlenecks currently hindering the translation of research into products and proposes future directions, including organoids and organ-on-a-chip technologies, AI-driven formulation optimization, and international multicenter clinical trials, with the aim of providing a systematic theoretical framework for the transition of MFH functional foods from “empirical application” to “precision interventions guided by evidence-based medicine”. Future research should prioritize randomized controlled trials, comprehensive safety assessments, evaluations of bioavailability, and standardized product validation to facilitate clinical translation.

## 2. Literature Search and Selection Strategy

This narrative review was conducted based on a systematic literature search strategy. PubMed/MEDLINE, Web of Science, CNKI, and Wanfang Data were searched from database inception to August 2026. In addition, the reference lists of relevant reviews were manually searched to supplement early classic literature that might not have been indexed in the databases due to the limitations of the search terms. The search strategy consisted of three groups of terms: (i) disease-related terms: “polycystic ovary syndrome” OR “PCOS” OR “polyendocrine metabolic ovarian syndrome” OR “PMOS”; (ii) MFH-related terms: “medicine-food homology” OR “medicinal and edible” OR “food-medicine dual use” OR “functional food” OR “dietary bioactive compounds”; and (iii) compound-related terms: “polyphenol” OR “flavonoid” OR “polysaccharide” OR “curcumin” OR “quercetin” OR “puerarin” OR “cinnamic acid” OR “glycyrrhizin” OR “naringin” OR “genistein” OR “daidzein”.

Inclusion criteria were peer-reviewed original research, systematic reviews, meta-analyses, randomized controlled trials, and preclinical studies that evaluated the effects of MFH-derived bioactive compounds on PMOS/PCOS-related metabolic, endocrine, inflammatory, OS, gut microbiota, or neuroendocrine outcomes. Exclusion criteria were conference abstracts, editorials, letters, comments, studies without full text, and studies not reporting relevant outcomes. After removing duplicate records, titles and abstracts were screened, followed by full-text assessment of potentially eligible studies.

**Figure 1:**
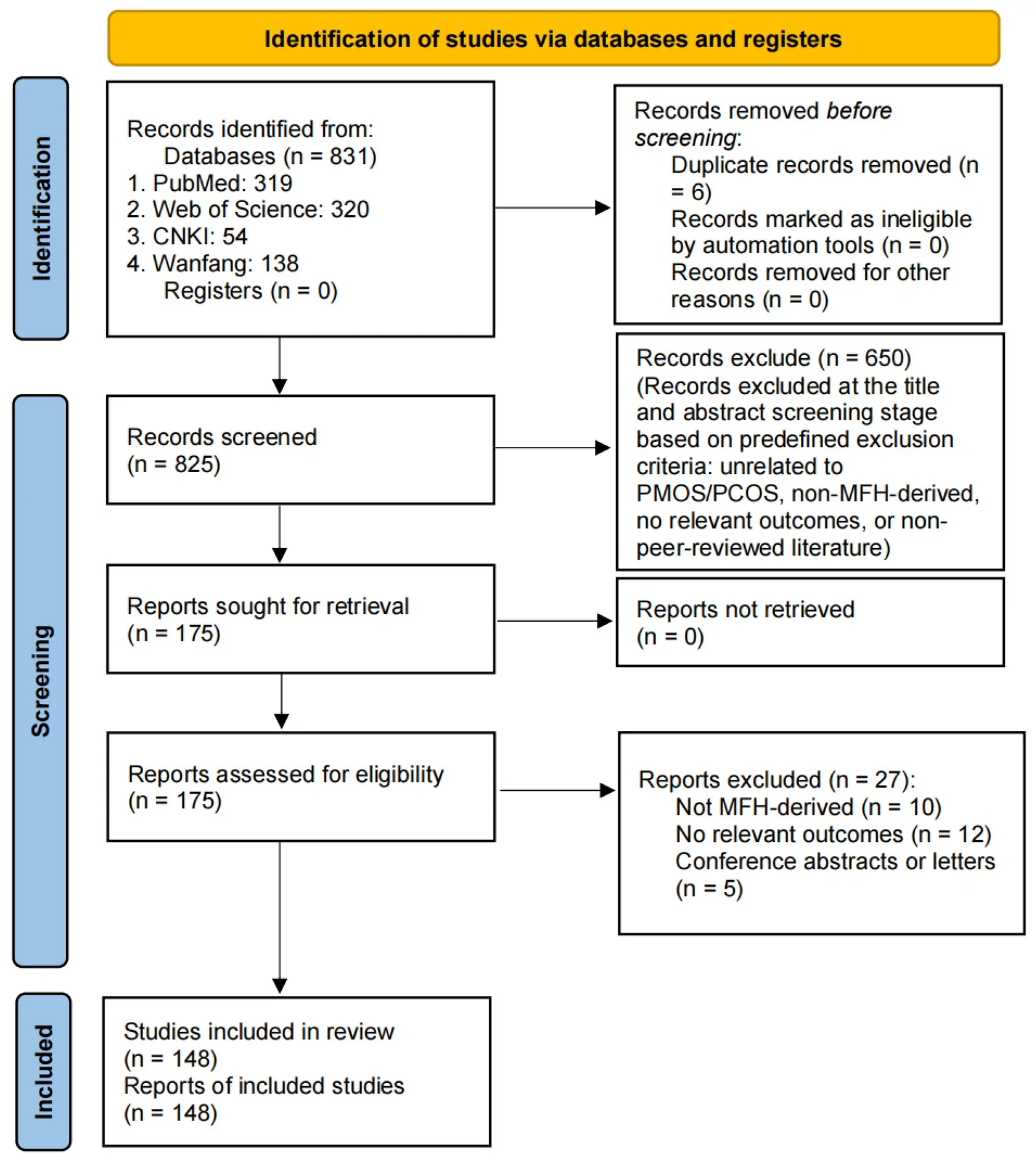
PRISMA 2020 flow diagram of the literature search and selection process.

A total of 831 records were initially identified; after removing duplicates, 825 records remained. Following title and abstract screening, 650 records were excluded, and 175 full-text articles were assessed, of which 148 studies were finally included. The screening process is detailed in the PRISMA flow diagram (Figure 1). Given that this is a narrative review, no meta-analysis was performed. Furthermore, due to significant heterogeneity among the included studies in terms of experimental design, interventions, and outcome measures, standardized quantitative risk-of-bias assessment tools (e.g., Cochrane RoB 2 or AMSTAR-2) were not applied. Instead, the methodological limitations of the included studies were integrated into the critical discussion throughout the manuscript, providing a qualitative presentation of the limitations and risk of bias of the available evidence.

## 3. Identification and Characterization of PMOS-Related Bioactive Compounds in MFH Herbal Medicines

### 3.1. Database of Major Traditional Chinese Medicinal Herbs with Dual Use as Food and Medicine

The MFH concept, based on the homology between food and medicine, provides a rich resource for dietary interventions and adjunctive therapies in PMOS. Commonly used MFH herbs include Chinese yam (*Dioscorea opposita*), goji berries (*Lycium barbarum*), hawthorn (*Crataegus pinnatifida*), poria (*Poria cocos*), coix seed (*Coix lacryma-jobi*), kudzu root (*Pueraria lobata*), mulberry leaf (*Morus alba*), cassia bark (*Cinnamomum cassia*), turmeric (*Curcuma longa*), and licorice (*Glycyrrhiza uralensis*). All herbs discussed in this review as MFH materials are included in this official catalog [27]. Their bioactive compounds may regulate glucose and lipid metabolism, improve IR, and exert anti-inflammatory and antioxidant effects in PMOS.

Representative components include puerarin from kudzu root, which has been reported to improve IR in ovarian granulosa cells and regulate sex hormone synthesis [28]; Chinese yam is rich in Dioscorea polysaccharide, which has immunomodulatory and antioxidant effects [29]; *Lycium barbarum* polysaccharide alleviates OS by regulating the Nrf2/HO-1 pathway [30]; cinnamic polyphenols and volatile compounds in cinnamon activate the AMPK pathway, improving insulin sensitivity in peripheral tissues [31]; and curcumin reduces chronic local inflammation in the ovaries by inhibiting NF-κB nuclear translocation [32]. Hawthorn flavonoids, coix seed esters, and mulberry leaf alkaloids (e.g., DNJ) also show potential in regulating lipid and glucose metabolism. Characterizing these components provides a basis for subsequent metabolomics- and network pharmacology-based screening.

### 3.2. Screening of Bioactive Compounds Based on Metabolomics and Network Pharmacology

#### 3.2.1. Applications of UPLC-MS/MS and GC-MS in Compound Identification

High-resolution mass spectrometry is essential for chemical profiling of complex MFH formulations. The combined use of UPLC-MS/MS and GC-MS enables the comprehensive characterization of non-volatile components with vastly different polarities (such as flavonoids, polysaccharides, and alkaloids) and volatile/semi-volatile components (such as terpenes and organic acids) in herbal materials, thereby providing a solid foundation of chemical information for subsequent activity-guided isolation and screening [33]. For instance, Shao et al. employed UHPLC-Q-Orbitrap HRMS to expeditiously profile 123 constituents in Pu Zhi Hui Ling Decoction, including flavonoids, terpenoids, and organic acids, thereby illustrating the utility of HRMS for the characterization of complex traditional Chinese medicine formulations [34].

High-resolution mass spectrometry platforms, including UHPLC-Q-Exactive Orbitrap and QTOF-MS, when coupled with UNIFI and Compound Discoverer, enable rapid identification of more than 100 compounds in MFH materials. For example, a related study employed a strategy combining UPLC-Q-TOF-MS/MS with GC-MS to successfully identify 262 candidate compounds from the traditional Chinese medicine formula “Shenxianshengmai” [35]. Building on this, the introduction of multivariate statistical analysis methods such as PCA and OPLS-DA can effectively identify differential metabolites between different processed preparations or different source herbal materials, thereby pinpointing potential quality markers (Q-markers) and bioactive components, and providing targeted evidence for compound quality control and research into the pharmacological basis of efficacy.

In biological matrices such as serum or tissue, serum pharmacochemistry coupled with UPLC-MS/MS distinguishes parent constituents from in vivo metabolites, thereby identifying the systemically exposed active components of MFH formulations. For example, Liu et al. used UPLC-Q-Exactive Orbitrap MS/MS combined with a serum pharmacochemistry strategy to identify 32 parent compounds and 36 metabolites in the serum of rats after administration of Yinchen Sini Decoction, and further screened for key bioactive compounds such as apigenin and naringin using network pharmacology. The above studies demonstrate that this strategy can effectively reveal the in vivo exposure profiles of TCM formulas, providing reliable technical support for the precise identification of bioactive compounds [36].

#### 3.2.2. Strategies for Constructing “Compound–Target-Pathway” Networks

Network pharmacology can systematically elucidate the multi-target synergistic mechanisms of MFH herbal medicines by constructing a multidimensional “drug-compound–target-disease” network. The standard workflow for this strategy typically includes the following steps: (1) Predicting targets for active compounds based on open databases such as TCMSP, SwissTargetPrediction, and SEA; (2) Integrating disease-related databases such as OMIM, DisGeNET, and GeneCards, as well as GEO differential gene expression data, to systematically collect PMOS-related target genes; (3) Constructing protein–protein interaction (PPI) networks and identifying core targets through topological parameter analysis; (4) Performing GO functional annotation and KEGG pathway enrichment analysis on core targets; (5) Using molecular docking (e.g., AutoDock) and molecular dynamics simulations to validate the binding affinity and complex stability between key constituents and core targets.

This strategy has been applied in numerous studies of PMOS-related traditional Chinese medicines and herbal medicines. For MFH-type herbal medicines, Taha et al. employed network pharmacology methods to analyze *Serenoa repens* extract, predicting that its major active components may regulate multiple PMOS-related pathways, including steroid hormone biosynthesis, endocrine resistance, and OS [37]. Abomughaid et al. integrated network pharmacology with molecular docking techniques and predicted that the active components of black cumin (*Nigella sativa*) may act on 53 PMOS common target genes; KEGG enrichment analysis suggested that ovarian steroidogenesis, IR, androgen signaling, and inflammatory pathways are its key regulatory modules [38]. Furthermore, Zhang et al., using a combined strategy of network pharmacology and experimental validation, screened the YJKL formula to identify five core targets, including AKT1, TP53, and TNF, and experimentally validated that their mechanisms of action primarily involve the AGE-RAGE, TNF, and HIF-1 signaling pathways. The above studies suggest that network pharmacology methods can effectively elucidate the multi-target regulatory networks of traditional Chinese medicine formulas and their active ingredients in complex diseases, providing important computational biology evidence for the subsequent identification of quality markers and the elucidation of mechanisms of action [39].

#### 3.2.3. Prediction of Potential Targets for Key Bioactive Compounds

Based on the topological analysis of the aforementioned “component–target-pathway” network, and in combination with KEGG enrichment results, it is possible to systematically predict the key nodes in the PMOS pathological network regulated by the active components of MFH. The computational results suggest that the potential targets of the candidate compounds are significantly enriched in four core pathways: insulin signaling (PI3K/AKT), energy sensing (AMPK), inflammatory response (NF-κB), and antioxidant defense (Nrf2/HO-1) [40]. Among the representative compounds, cinnamic acid and curcumin are predicted to be potential activators of the AMPK pathway; puerarin and Dioscorea polysaccharides are predicted to primarily target the PI3K/AKT axis to improve insulin sensitivity, while Lycium polysaccharides and hawthorn flavonoids are predicted to exert synergistic antioxidant and anti-inflammatory effects by activating Nrf2/HO-1 and inhibiting NF-κB nuclear translocation [40]. Furthermore, computational analysis predicts that licorice flavonoids may be involved in regulating the expression of androgen synthases such as CYP17A1, suggesting a potential role in the local metabolism of steroid hormones in the ovary [41]. These predictive results provide a clear direction for subsequent experimental validation; the biological functions of each pathway and their specific molecular evidence will be systematically discussed in Section 4. These computational predictions should be regarded as hypothesis-generating and require experimental validation; they should not be interpreted as established mechanisms.

**Figure 2:**
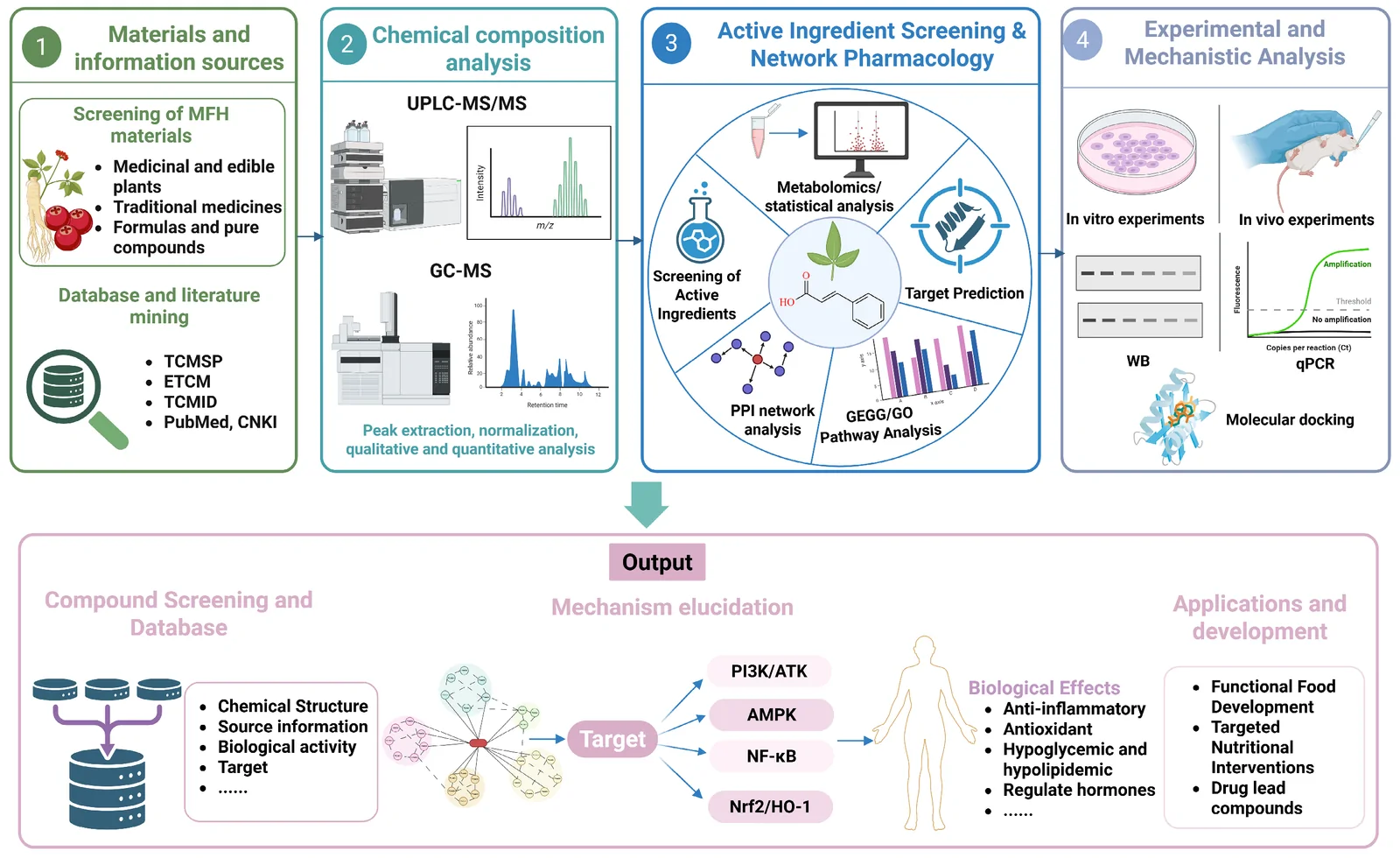
Integrated screening and mechanistic prediction of bioactive compounds from MFH. The workflow integrates multi-dimensional approaches, including chemical profiling via UPLC-MS/MS and GC-MS, multivariate statistical analysis, database-driven active ingredient screening, target prediction, and network analysis (PPI, KEGG/GO), followed by in vitro and in vivo experimental validation (Western blot, qPCR). The identified core pathways (PI3K/AKT, AMPK, NF-κB, and Nrf2/HO-1) underpin the potential biological actions of MFH-derived compounds against IR, inflammation, and OS, offering a comprehensive strategy for the discovery of food bioactive ingredients with pharmacological applications.

Given this, the chemical and computational analyses above identify candidate compounds and predict potential targets (Figure 2), but they do not establish mechanisms. The next section therefore examines the mechanistic evidence linking these compounds to PMOS pathophysiology, explicitly distinguishing direct PMOS evidence from indirect mechanistic evidence.

## 4. Mechanisms of Action: Linking Food Bioactives to PMOS Pathophysiology

Based on the candidate compounds and predicted pathways identified above, this section evaluates the mechanistic evidence for MFH bioactives in PMOS. Evidence is classified as direct PMOS evidence, indirect mechanistic evidence, or computational prediction, so that predicted mechanisms are not presented as established ones.

The pathophysiological mechanisms of PMOS involve the interplay of multiple factors, including IR, hyperandrogenism, chronic low-grade inflammation, OS, and neuroendocrine dysfunction. The active constituents of MFH, characterized by multi-target and multi-pathway regulatory activities, may furnish a plausible molecular basis for a comprehensive intervention in PMOS; however, their clinical efficacy, long-term safety, bioavailability, and product feasibility remain to be established. This chapter systematically elucidates the key molecular mechanisms by which MFH bioactive compounds intervene in the pathophysiological network of PMOS, covering five aspects: improvement of glucose and lipid metabolism, regulation of ovarian steroid synthesis, alleviation of inflammation and OS, modulation of the gut microbiota–ovarian axis, and maintenance of homeostasis in the HPO axis. To ensure accurate interpretation of the evidence, the following model classification is adopted.

When citing individual studies, the original terminology and diagnostic context are retained; for example, “letrozole-induced PCOS rat model (reported as PCOS in the original study)” is used rather than simply “PMOS model.” Accordingly, preclinical evidence is categorized into two groups based on model relevance. This section categorizes preclinical evidence into two groups based on model relevance. The first group consists of PMOS-specific models, including but not limited to rodent models induced by commonly used PMOS agonists (such as letrozole, DHEA, DHT, estradiol valerate, clomiphene, etc.), models combined with metabolic stress such as a high-fat diet, PMOS-related cellular models (such as KGN cells and primary granulosa cells), and patient-derived samples from PMOS patients (such as cells, tissues, and feces, which may be labeled as PCOS in historical literature). These findings provide direct evidence for PMOS. The second category consists of non-PMOS metabolic models, including but not limited to high-fat diet-induced obesity, streptozotocin-induced type 2 diabetes, the HepG2 hepatic steatosis model, and high-fat diet-fed zebrafish. These results serve only as mechanistic references and cannot be interpreted as direct evidence of efficacy for PMOS. The model type is clearly labeled throughout the text, and conclusions are limited accordingly. A comprehensive summary of these preclinical studies, detailing their models, model relevance, evidence levels, administration methods, and mechanisms of action, is presented in Table 1.

### 4.1. Improving IR and Glucose and Lipid Metabolism

IR is one of the core pathological features of PMOS. Clinical studies show that approximately 75% of non-obese PMOS patients and 95% of obese patients exhibit IR. The molecular basis of this lies in increased serine phosphorylation and decreased tyrosine phosphorylation of the insulin receptor and IRS1, leading to impaired downstream signal transduction [42]. The PI3K/AKT pathway is central to insulin signaling; after insulin binds to the receptor, IRS is recruited to the receptor, activating PI3K and converting PIP2 to PIP3, which binds to the PH domain of AKT and, with the assistance of PDK1 and mTORC2, phosphorylates AKT at Thr308 and Ser473. Activated AKT subsequently promotes the translocation of GLUT4 from the cytoplasm to the cell membrane, mediating insulin-stimulated glucose uptake. In the adipose tissue of patients with PMOS, GLUT4 expression is reduced, and there is no compensatory increase in GLUT1 [43].

At the same time, AMPK, acting as the master switch of energy metabolism, also plays a key regulatory role in PMOS-IR. Studies have found that reduced circulating adiponectin levels in the skeletal muscle of PMOS patients can impair AMPK activity, while the response of pyruvate dehydrogenase to insulin stimulation is weakened; together, these two factors drive the onset and progression of IR [44]. Unlike PI3K/AKT-mediated insulin signaling, AMPK primarily responds to cellular energy stress by phosphorylating downstream substrates to promote glucose uptake and fatty acid oxidation, thereby forming a complementary metabolic regulatory network with PI3K/AKT [45].

Based on these two core pathways, bioactive compounds derived from MFH materials have become an important source for drug screening to address PMOS-related glucose and lipid metabolic disorders, owing to their multi-target regulatory effects and generally favorable safety profiles in preclinical studies, although long-term safety data are limited. In recent years, active components from typical MFH materials, such as curcumin, puerarin, Dioscorea polysaccharides, and trans-cinnamic acid, have demonstrated clear effects in improving IR and regulating lipid homeostasis in PMOS and related metabolic disorder model systems. Regarding their mechanisms of action, current evidence suggests that these compounds primarily exert their effects through two pathways: first, by directly targeting PI3K/AKT or AMPK signaling nodes to correct insulin signal transduction and energy metabolism; and second, by improving the pathological microenvironment of IR through indirect pathways such as the gut barrier–inflammation axis. The specific molecular mechanisms and preclinical evidence for each of these compounds will be detailed in subsequent chapters.

#### 4.1.1. Preclinical Studies

Regarding curcumin, the Yang-induced PCOS mouse model confirmed that this compound can reduce intestinal mucosal permeability and plasma lipopolysaccharide (LPS) levels by upregulating the expression of the colonic tight junction proteins ZO-1 and occludin, thereby inhibiting the ovarian TLR4/MyD88/NF-κB signaling pathway, alleviating systemic and ovarian inflammation, and significantly improving insulin sensitivity, suggesting that curcumin can improve PCOS-related IR through anti-inflammatory mechanisms [46]. In a letrozole-induced PCOS rat model, Zheng et al. [47] further found that curcumin inhibits the excessive activation of the IRS1/PI3K/AKT signaling pathway by upregulating PTEN expression, thereby restoring GLUT4-mediated glucose uptake. This significantly reduces fasting blood glucose, insulin levels, and HOMA-IR, and improves ovarian morphology and hormonal dysregulation, suggesting that this compound may also exert its beneficial effects by directly correcting insulin signaling. These two mechanisms are complementary and collectively support the potential value of curcumin in the treatment of PCOS.

Regarding puerarin, Xu et al. [48] used a high-fat, high-fructose diet-induced obese rat model and HepG2 cells stimulated with free fatty acids (FFAs) combined with fructose to demonstrate that puerarin, by activating the AMPK and PI3K/Akt signaling pathways, inhibits the expression of SREBP-1c, FAS, and promotes GLUT4-mediated glucose uptake, thereby significantly improving IR and hepatic lipid deposition, indicating that puerarin has therapeutic potential in correcting glucose and lipid metabolism disorders. Building on these findings, Pham et al. [49] used a high-glucose-induced HepG2 cell steatosis model to further reveal that puerarin, by activating GPER-coupled Ca^2+^/CaMKKβ and SIRT1/LKB1 signaling cascades, enhances AMPK phosphorylation, thereby inhibiting SREBP-1c/FASN-mediated lipid synthesis and upregulating ATGL to promote lipolysis, significantly reducing lipid accumulation in hepatocytes. This suggests that puerarin’s activation of AMPK depends on upstream GPER-coupled calcium signaling and the SIRT1/LKB1 signaling cascade. It should be noted that the above model is not specific to PMOS and cannot reproduce the ovarian and hyperandrogenic features of PMOS; therefore, it serves only as a reference for understanding the underlying mechanisms and does not constitute direct evidence of the model’s efficacy in treating PMOS.

Furthermore, Feng et al. [50] used a mouse model of T2DM induced by a high-fat diet combined with streptozotocin (STZ) to demonstrate that CYPB, an acidic polysaccharide derived from Chinese yam, enhances the expression of InsR, PI3K, Akt, and GLUT4 protein expression, and by inhibiting FoxO3-mediated gluconeogenesis, thereby significantly reducing fasting blood glucose, improving IR, and increasing hepatic glycogen synthesis. This suggests that Dioscorea polysaccharides have potential value in the management of IR. Research by Jia et al. [51] demonstrated that trans-cinnamic acid activates the AMPK-mTOR signaling axis by directly binding to AMPKα1 (Arg133/His134), thereby inhibiting the expression of lipogenesis-related genes such as SREBP1, PPARγ, and FAS, while simultaneously enhancing glycolysis and the TCA cycle, significantly reducing high-fat diet-induced hepatic lipid accumulation. Given that AMPK is a core target for improving IR, this study provides direct evidence that the active components of cinnamon improve glucose and lipid metabolism disorders by activating the AMPK pathway. However, neither of the two studies mentioned above was directly validated in a PCOS model. Although the PI3K/Akt and AMPK pathways involved are key regulatory nodes in PCOS IR and can serve as targets for future research, their results represent only indirect evidence of the underlying mechanisms.

In summary, curcumin has been directly evaluated in PCOS models, whereas puerarin, CYPB, and trans-cinnamic acid have primarily been studied in models of obesity, T2DM, or hepatic steatosis. The findings regarding the latter should be regarded as hypothesis-generating and must be validated in PCOS/PMOS models before they can be translated into PMOS-specific recommendations.

#### 4.1.2. Clinical Studies

Zohrabi et al. [52] conducted a 12-week randomized, double-blind clinical trial involving 104 women with PCOS-related infertility. These findings suggest that the DASH diet in combination with curcumin (500 mg twice daily) was associated with significant reductions in insulin concentrations and HOMA-IR relative to controls, whereas fasting blood glucose and QUICKI remained unchanged. Collectively, these results suggest that the DASH diet with curcumin may ameliorate PCOS-associated IR and hyperinsulinemia, though confirmation in larger trials is warranted. Moslehi et al. [53] evaluated the effects of various nutritional interventions on PCOS through an umbrella review of 28 RCTs and found that curcumin supplementation reduces fasting blood glucose, fasting insulin, and HOMA-IR in women with PCOS with moderate certainty, while probiotics/probiotic/prebiotic combinations can reduce total testosterone, and inositol can increase ovulation rates. This suggests that active ingredients from food–medicine dual-use sources, such as curcumin, may have potential in improving PCOS-related IR and glucose–lipid metabolism disorders; however, the certainty of evidence remains limited by small sample sizes, short intervention periods, inconsistent dosing, and low or very low certainty. A meta-analysis of 12 randomized controlled trials reported that cinnamon supplementation was associated with significant reductions in fasting plasma glucose, HOMA-IR, total cholesterol, and LDL-C levels in women with PCOS, suggesting that cinnamon’s bioactive constituents may confer beneficial effects on PCOS by improving IR and lipid metabolism. However, additional randomized controlled trials are warranted to confirm these findings [54]. Salman Al Anei et al. [55] conducted a 3-month randomized clinical trial involving 75 women with PCOS; the results showed that Diane-35 combined with licorice extract (450 mg, twice daily) significantly reduced HOMA-IR, fasting insulin, total cholesterol, and IL-1β levels compared to Diane-35 alone, while increasing HDL and total antioxidant capacity (TAC). This indicates that licorice, as a food–medicine dual-use plant, has potential as an adjunctive therapy for improving PCOS-related IR and lipid metabolism disorders. However, larger trials with longer follow-up and standardized dosing are needed. In addition to the studies discussed above, a comprehensive summary of the clinical studies across Section 4.1.2 to Section 4.5.2 is presented in Table 2.

### 4.2. Regulation of the Local Ovarian Microenvironment and Steroid Synthesis

In the theca cells of women with PCOS, the expression and enzymatic activity of CYP11A1 and CYP17A1 are significantly elevated; this characteristic is considered the core enzymatic basis driving excessive androgen production and hyperandrogenism [56,57]. Single-cell RNA sequencing studies have further revealed that the differential expression of CYP11A1 and CYP17A1 in PCOS theca cells is primarily driven by the regulatory networks of the transcription factors SREBF1 and GATA6 [58]. In addition, the expression of hydroxysteroid dehydrogenases such as HSD3B2 and HSD17B is also altered in PCOS theca cells, contributing to the overall upregulation of the androgen synthesis pathway [59]. This abnormally hyperactive steroidogenic microenvironment not only directly impairs granulosa cell function and follicular development but also serves as a key hub where IR and chronic inflammation interact to exacerbate the hyperandrogenic state [60].

Given the pivotal role of the aforementioned steroid synthesis pathways in the pathogenesis of PCOS, research on the active components of MFH in regulating the local ovarian microenvironment and androgen metabolism has made significant progress in recent years, encompassing both preclinical mechanism analysis and clinical efficacy validation. The following section will systematically review the mechanisms of action and therapeutic potential of MFH’s active components in modulating the local ovarian microenvironment and steroid synthesis in PCOS from both preclinical and clinical research perspectives.

#### 4.2.1. Preclinical Studies

Using a PCOS rat model induced by letrozole combined with a high-fat diet and a DHT-induced KGN cell model, Zhang et al. [61] demonstrated that glycyrrhizic acid, glycyrrhizin, genistein, and quercetin in Erchen Decoction can regulate the steroid hormone synthesis pathway by downregulating CYP17A1 and HSD11B1 and upregulating AKR1D1 and HSD11B2, thereby reducing serum testosterone, LH, and AMH levels, restoring estradiol levels, and alleviating ovarian histopathological damage. Li et al. [62] further demonstrated using a letrozole- or DHEA-induced PCOS mouse model that glycyrrhizin (20 mg/kg) can downregulate the expression of Cyp17a1, Cyp19a1, Fshr, and Runx2—genes associated with steroid synthesis and follicular development—to reduce serum total testosterone and dehydroepiandrosterone sulfate levels, decrease the number of cystic follicles, and increase the number of corpus luteum. Yang et al. [63] used a letrozole-induced PCOS rat model to demonstrate that an ethanol extract of licorice (300 mg/kg) can improve ovarian tissue morphology and hormonal imbalance by upregulating the expression of Cyp11a1 and Ptgs2 in the ovary, restoring serum FSH levels, and reducing the LH/FSH ratio. The above studies indicate that licorice and its active component, glycyrrhizin, have therapeutic potential in improving the local ovarian microenvironment and correcting androgen synthesis disorders in PCOS by targeting key enzymes involved in ovarian steroid synthesis and gonadotropin signaling pathways.

Furthermore, using a letrozole-induced PCOS rat model, Rajan et al. [64] found that soy isoflavones (50, 100 mg/kg) can reduce testosterone levels by inhibiting ovarian 3β-HSD and 17β-HSD activity, while simultaneously alleviating OS and restoring normal ovarian follicular structure, thereby improving estrous cycle disorders and polycystic-like ovarian changes. Research by Taghipour et al. [65] in a letrozole-induced PCOS rat model showed that the quercetin-cyclodextrin complex (75 mg/kg) can improve the ovarian microenvironment and steroid synthesis homeostasis by downregulating ovarian CYP11A and CYP17A gene expression, upregulating CYP19A expression, reducing OS, and restoring normal follicular development and corpus luteum formation. Using a DHEA-induced PCOS mouse model and primary granulosa cells, Luo et al. [66] found that genistein (8.75 mg/kg) can restore the expression of steroid synthesis genes such as Star, Cyp11a1, Hsd3b1, and Cyp19a1, improve serum T, E2, P4, and LH/FSH ratios, reduce the number of cystic follicles, and restore the estrous cycle. The above studies indicate that flavonoid components derived from MFH, such as soy isoflavones, quercetin, and genistein, can exert therapeutic effects by targeting the activity or gene expression of steroid synthases and alleviating OS, thereby modulating the local ovarian steroid synthesis microenvironment in PCOS.

Shen et al. [67] used a rat model of PCOS induced by a high-fat diet combined with letrozole and a DHT-induced KGN granulosa cell model to demonstrate that the Pinellia-Chenpi herbal combination (1.8 g/kg), by downregulating CYP17A1 and CYP19A1 expression and upregulating AKR1C3 and HSD17B1, regulating the steroid biosynthesis pathway, thereby reducing serum testosterone and restoring E2, FSH, LH, and AMH levels, decreasing the number of cystic follicles and increasing the number of corpus luteum, and improving ovarian tissue morphology and the estrous cycle. These findings suggest that this herbal combination, with Chenpi serving as a food–medicine dual-use ingredient, has application potential in modulating the ovarian steroid synthesis microenvironment in PCOS.

#### 4.2.2. Clinical Studies

In a randomized, double-blind, controlled trial involving 66 overweight/obese women with PCOS, Hooshmandi et al. [68] reported that licorice extract (1.5 g/day for 8 weeks) combined with a low-calorie diet significantly increased serum FSH levels, reduced the LH/FSH ratio and total testosterone concentration, and effectively improved depressive symptoms, sleep quality, and appetite. This suggests that licorice extract, as an active component of MFH, has therapeutic potential in regulating ovarian steroid synthesis and the neuroendocrine microenvironment in PCOS. However, the sample size was relatively small, and the intervention duration was short; further trials are needed. A preliminary randomized, double-blind, controlled trial conducted by Majeed et al. [69] involving 60 women with PCOS further demonstrated that a compound herbal tea containing marjoram and cinnamon (twice daily for 3 months) significantly increased FSH levels and decreased AMH concentrations, suggesting that cinnamon-based herbal formulations derived from food and medicine can improve PCOS-related endocrine disorders by regulating the local ovarian microenvironment for follicular development and steroid hormone synthesis pathways. Nevertheless, this preliminary, small-scale trial necessitates confirmation of these findings through larger randomized controlled trials.

### 4.3. Anti-Inflammatory Effects and Alleviation of OS

OS and chronic low-grade inflammation mutually drive each other in PMOS, constituting a key link in disease progression. Antioxidants exert their mitigating effects through dual pathways: inhibiting the NF-κB/NLRP3 inflammasome pathway and activating the Nrf2/HO-1 antioxidant defense system.

In the PMOS state, hyperglycemia and FFAs induce excessive ROS production, activating the IκB kinase (IKK) complex, which promotes NF-κB nuclear translocation and initiates the transcription of pro-inflammatory factors such as TNF-α and IL-6 [70]. Concurrently, ROS act as a secondary signal to promote NLRP3 inflammasome assembly, activate caspase-1, and release IL-1β and IL-18, further exacerbating local ovarian inflammation and disrupting insulin signaling pathways [71]. Antioxidants can effectively block this inflammatory cascade by scavenging ROS or interfering with NF-κB nuclear translocation.

Nrf2 is a central transcription factor in cellular antioxidant defense. OS promotes the dissociation of Nrf2 from Keap1 and its translocation into the nucleus, where it binds to the antioxidant response element (ARE) to initiate the expression of downstream antioxidant enzymes such as HO-1, NQO1, and glutathione S-transferase [72]. HO-1 exerts anti-inflammatory and cytoprotective effects by degrading heme to produce biliverdin and carbon monoxide [73]. Clinical studies have shown that an increase in total TAC following antioxidant intervention occurs concurrently with a decrease in CRP, suggesting a functional coupling between Nrf2 pathway activation and inflammation suppression [74].

In summary, antioxidants synergistically alleviate OS and inflammation in PMOS through the aforementioned dual pathways, providing a molecular basis for their clinical application.

#### 4.3.1. Preclinical Studies

Curcumin possesses dual pharmacological activities—anti-inflammatory and antioxidant. Two independent studies examined these effects from two distinct perspectives: local OS in the ovaries and inflammation in the gut–ovarian axis, respectively. Zhang et al. [75] used a DHEA-induced PCOS rat model and demonstrated that daily oral administration of curcumin reduced local ovarian OS by upregulating ovarian PPAR-γ expression, lowering ROS and MDA levels, and increasing SOD, GPx, and GSH levels, thereby directly alleviating local ovarian OS and improving sex hormone dysregulation, IR, and cystic ovarian morphology; Yang et al. [46] administered daily oral curcumin to a DHEA-induced PCOS mouse model and found that the compound repairs the intestinal mucosal barrier and reduces permeability by upregulating ZO-1 and Occludin expression in the colon, thereby decreasing plasma LPS translocation. This, in turn, inhibits the activation of the ovarian TLR4/MyD88/NF-κB signaling pathway, downregulating IL-17A, IL-6, and TNF-α while upregulating IL-10, ultimately alleviating systemic and local ovarian inflammation and improving PCOS-related symptoms. These results indicate that curcumin, derived from the Curcuma genus, has potential therapeutic value in the treatment of PCOS. Similarly, regarding the antioxidant and anti-inflammatory mechanisms of quercetin in PCOS, existing evidence reveals its action characteristics from two perspectives: hepatic OS/epigenetic regulation and the ovarian inflammatory microenvironment. Jafari-Khorchani et al. [76] used a DHEA-induced PCOS rat model and found that after oral administration of quercetin, the compound upregulated liver SOD, CAT, GPx, and GR activity as well as GSH levels, reduced MDA content, and downregulated ovarian DNMT1 and DNMT3a expression. This alleviated OS and epigenetic dysregulation, restored serum zinc levels, and improved ovarian morphology; Wang et al. [77] used a DHEA-induced PCOS rat model and administered quercetin derived from hawthorn via oral gavage. They found that this active compound, by inhibiting the ovarian TLR4/NF-κB signaling pathway, downregulated the expression of p22phox, OX-LDL, as well as IL-1β, IL-6, and TNF-α, and by blocking NF-κB p65 nuclear translocation, thereby improving the inflammatory microenvironment and IR, and restoring the estrous cycle and ovarian morphology. These results indicate that quercetin derived from food–medicine plants such as hawthorn has potential therapeutic value in the treatment of PCOS.

#### 4.3.2. Clinical Studies

In a systematic review and meta-analysis incorporating 7 randomized controlled trials involving a total of 447 women with PCOS, Shen et al. [78] reported that curcumin supplementation was associated with significant reductions in CRP levels in patients (WMD −0.785, 95% CI −1.553 to −0.017), while simultaneously improving metabolic parameters such as body mass index, fasting blood glucose, insulin, and IR indices. At the mechanistic level, curcumin—the primary polyphenolic component of turmeric—exerts its anti-inflammatory effects by inhibiting the NF-κB signaling pathway and downregulating the expression of inflammatory factors such as IL-6. This ameliorates the chronic low-grade inflammatory milieu linked to PCOS and facilitates the resolution of metabolic dysregulation, providing preliminary evidence for turmeric’s potential as an MFH material in adjunctive management of PCOS; however, the certainty of evidence remains limited by small sample sizes, brief intervention durations, inconsistent dosing, and low to very low certainty.

In addition to turmeric, researchers have also extended the development of food–medicine dual-use plant resources to the exploration of bioactive compounds in non-edible parts or medicinal materials not listed in the official catalog; the mulberry tree serves as a typical example. Although mulberry branches (SZ-A) have not yet been included in the national directory of food–medicine dual-use materials, its leaves (mulberry leaves) and fruits (mulberries) are both legally recognized food–medicine dual-use materials. Furthermore, modern chemical analysis shows that different parts of the mulberry tree contain active alkaloid components, represented by 1-deoxynojirimycin (DNJ) [79,80]. In the same study, Liu et al. [81] integrated three levels of research—animal experiments, cellular mechanisms, and clinical trials—to systematically evaluate the therapeutic effects of total alkaloids from SZ-A on PCOS. At the animal and cellular levels, SZ-A significantly inhibited the phosphorylation of NF-κB and IκBα in ovarian tissue, reduced the activation of inflammatory pathways, and improved mitochondrial dysfunction and granulosa cell apoptosis. Building on these findings, the study further confirmed through a randomized, double-blind, placebo-controlled trial involving 128 women with PCOS that SZ-A (300 mg/day for 24 weeks) significantly reduced the free androgen index (FAI), increased SHBG levels, and restored regular menstruation in 34.92% of participants (compared to 7.69% in the placebo group), while also effectively improving BMI and insulin secretion profiles. By systematically validating the anti-inflammatory mechanisms from preclinical studies through to clinical efficacy, this research provides multi-level evidence for the use of active components derived from mulberry trees in the treatment of PCOS. It also suggests that the non-edible parts of medicinal and edible plants serve as an important repository for identifying lead compounds with anti-inflammatory activity.

### 4.4. Regulation of the Gut–Ovary Axis

Gut microbiota dysbiosis is one of the key pathological features of PMOS; both PMOS patients and animal models exhibit reduced α-diversity of the microbiota, altered abundances of specific bacterial genera, and impaired intestinal barrier function [82]. Short-chain fatty acids (SCFAs), metabolites derived from the gut microbiota, particularly acetic acid, propionic acid, and butyric acid, are key signaling molecules linking the gut microbiome to the host’s metabolic and endocrine networks [83]. Mechanistically, SCFAs participate in the pathological regulation of PMOS primarily through the following three pathways:

First, SCFAs exert epigenetic regulation by inhibiting histone deacetylase (HDAC) activity. Studies have shown that acetate can reduce HDAC2 levels, thereby alleviating local OS (by lowering MDA and increasing GSH) and inflammatory responses (by inhibiting TNF-α and NF-κB expression) in adipose tissue and the ovaries, thus improving IR and hyperandrogenism in rats with PCOS [84].

Second, SCFAs regulate energy metabolism and inflammatory responses by activating G protein-coupled receptors (such as GPR41/GPR43). This pathway promotes glucagon-like peptide-1 (GLP-1) secretion, improves insulin sensitivity, and simultaneously inhibits the release of inflammatory factors from adipose tissue, thereby indirectly alleviating the local inflammatory microenvironment in the ovaries [85].

Third, SCFAs reduce systemic translocation of LPS by maintaining the integrity of the intestinal mucosal barrier. Once LPS enters the systemic circulation via the portal vein, it can activate the TLR4/NF-κB signaling pathway in the ovaries and adipose tissue, driving the release of pro-inflammatory factors such as IL-6 and TNF-α, which in turn upregulate the expression of androgen-synthesizing enzymes such as CYP17A1, thereby exacerbating hyperandrogenism [82]. By upregulating the expression of intestinal epithelial tight junction proteins (ZO-1, Occludin), SCFAs repair intestinal barrier function, thereby interrupting the aforementioned pathological cascade of “leaky gut–endotoxemia–ovarian inflammation–hyperandrogenism” [86]. Furthermore, microbial metabolites, such as flavonoid degradation products like 3,4-dihydroxyphenylacetic acid (DHPAA), have been shown to lower AMH levels by inhibiting the BMP signaling pathway and improve the reproductive phenotype in PMOS, suggesting that the metabolism of MFH flavonoids by the gut microbiota may constitute one of the potential mechanisms underlying its regulation of the gut–ovary axis [87].

#### 4.4.1. Preclinical Studies

Using a clomiphene-induced PCOS rat model, Li et al. [88] found that Monk fruit extract (MGE) can enrich beneficial bacterial genera such as Akkermansia and Parasutterella in the gut, restore levels of anti-inflammatory metabolites such as butyrate, and inhibit the activation of the ovarian NF-κB/NLRP3 inflammatory pathway, thereby improving estrous cycle disorders and reducing serum testosterone and insulin levels. This indicates that MGE, as an active component of the food–medicine plant Monk fruit, exerts anti-inflammatory effects by regulating the gut microbiota–ovarian axis, thereby alleviating the pathological progression of PCOS. He et al. [89] used a mouse model of PCOS induced by clomiphene combined with a high-fat diet and a DHT-induced KGN cell fibrosis model to demonstrate that quercetin can enrich the gut genus Akkermansia and promote the production of SCFAs such as acetic acid, thereby regulating the TGF-β signaling pathway by inhibiting SMAD3 phosphorylation and upregulating SMAD7 expression, which in turn alleviates ovarian fibrosis, restores the estrous cycle, and improves insulin sensitivity. This indicates that quercetin, as an active flavonoid component widely distributed in food–medicine plants such as hawthorn, exerts an anti-fibrotic effect by modulating the gut microbiota–SCFAs–ovarian axis. Liu et al. [90] transplanted feces from PCOS patients before and after ZQLHR treatment into DHEA-induced PCOS mice and found that in mice receiving feces from the treatment group, the number of cystic follicles in the ovaries decreased while the number of corpus luteum increased; serum T, E2, and LH levels were significantly reduced, FSH levels were elevated, and butyrate levels increased and were significantly correlated with changes in sex hormones. This indicates that the ZQLHR compound formula, centered on medicinal and edible herbs such as coix seed, licorice, and peach kernel, can improve endocrine disorders in PMOS by regulating the gut microbiota–butyrate metabolism–ovarian axis.

#### 4.4.2. Clinical Studies

Liu et al. [90] conducted a before-and-after controlled study involving 25 patients with PCOS and found that 6 months of treatment with ZQLHR significantly reduced serum total testosterone, LH, and the LH/FSH ratio, while increasing the abundance of butyrate-producing bacteria (Lachnospira, Faecalibacterium) and reducing the levels of opportunistic pathogens such as Escherichia and Shigella. Serum metabolomics revealed significant enrichment of the butyrate metabolic pathway, which may ameliorate hyperandrogenism in PCOS by modulating the gut microbiota–butyrate axis; however, these findings are preliminary and require validation in larger randomized controlled trials. In a randomized controlled trial involving 25 PCOS patients, Wang et al. [91] found that a 12-week intervention with a high-fiber diet (containing coix seed, Chinese yam, goji berries, white kidney beans, lotus seeds, and other food–medicine dual-use ingredients) for 12 weeks was associated with reductions in the LH/FSH ratio and testosterone levels, alleviated chronic inflammation, and improved brain–gut peptide secretion; the combination with acarbose yielded even better results; 16S rRNA sequencing revealed that the intervention group exhibited an enrichment of Bifidobacterium and Lactobacillus co-abundance groups (CAGs), and this enrichment of CAGs was significantly negatively correlated with the LH/FSH ratio, testosterone, HOMA-IR, and α-AGP, suggesting that a high-fiber diet centered on MFH ingredients may improve hyperandrogenism and IR in PMOS by modulating the gut microbiota–ovarian axis; however, this preliminary finding from a small, short-term trial requires confirmation in larger randomized controlled trials. Although both of the aforementioned clinical studies used compound formulations or dietary regimens rather than single herbal ingredients as the intervention, the core components, including coix seed, licorice, Chinese yam, and goji berries, constitute the foundation of active compounds. Their effects in regulating gut microbiota structure and restoring metabolic homeostasis precisely confirm the potential value of food–medicine dual-use materials as components of dietary interventions in the adjunctive treatment of PMOS, and also provide a clinical direction for subsequent screening of active ingredients and mechanism analysis based on single food–medicine dual-use herbs.

### 4.5. Regulation of the HPO Axis and Neuroendocrine Homeostasis

The core neuroendocrine feature of PMOS is dysfunction of the HPO axis, manifested by an increased pulse frequency of GnRH, which in turn leads to a relative predominance of LH over follicle-stimulating hormone (FSH) and an elevated LH/FSH ratio. This abnormality is closely associated with dysfunction of kisspeptin neurons in the hypothalamus [92]. As a key upstream regulator of GnRH neurons, kisspeptin stimulates GnRH release by binding to the GPR54 receptor, while differential expression of the Kiss1 gene in the arcuate nucleus (ARC) and the anterior ventromedial periventricular nucleus (AVPV)—two hypothalamic nuclei—mediates negative and positive feedback regulation of estrogen, respectively [93]. Phytoestrogen-like effects (selective estrogen receptor modulation) represent another important mechanism in HPO axis regulation. Flavonoids (such as genistein and daidzein), due to their structural similarity to 17β-estradiol, can bind to estrogen receptors α and β, selectively activating or antagonizing estrogen signaling pathways in specific tissues [94]. This effect has dual significance in the pathophysiology of PMOS: on the one hand, by activating ERβ, it enhances estrogenic negative feedback signaling, inhibits abnormally high-frequency GnRH pulses, and thereby reduces the LH/FSH ratio and excessive ovarian androgen synthesis; on the other hand, it upregulates hepatic SHBG synthesis, reducing the bioavailability of free testosterone [95].

#### 4.5.1. Preclinical Studies

At the animal experiment level, multiple studies have validated the regulatory effects of bioactive components derived from food and medicine on the kisspeptin–GnRH neuronal network of the HPO axis from various perspectives. Hu et al. [96] used a mouse model of PCOS induced by prenatal DHT exposure and found that 4 weeks of oral administration of crocin restored kisspeptin expression in the AVPV and reduced kisspeptin levels in the ARC, thereby correcting abnormal GnRH/LH pulsations, reducing the LH/FSH ratio and serum testosterone levels, and improving the estrous cycle and ovarian morphology. This indicates that crocin, as an active component of the food–medicine plant saffron (*Crocus sativus*), can improve neuroendocrine disorders in PCOS by regulating the hypothalamic kisspeptin–GnRH neuronal network. In contrast, Peng et al. [97] used a rat model of PCOS-IR induced by clomiphene combined with a high-fat diet and found that oral administration of total flavonoids from Eucommia Ulmo ides leaves for 21 days not only downregulated the expression of Kiss1, IGF-1, and the androgen receptor (AR) in the arcuate nucleus of the hypothalamus but also upregulated Kiss1 expression in the pituitary and ovaries while reducing AR levels. At the same time, it activated the ovarian PI3K/AKT pathway and upregulated GLUT4 expression, thereby improving sex hormone secretion disorders and IR while regulating Kisspeptin signaling. This indicates that total flavonoids from *Eucommia ulmoides* leaves, as the active components of this food–medicine dual-use plant, exert a protective effect through synergistic intervention of the Kisspeptin signaling network in the HPO axis and the ovarian insulin signaling pathway. Furthermore, Habibi et al. [98] studied an estradiol valerate-induced PCOS rat model and found that naringin (50 mg/kg, intraperitoneal injection, for 14 days) significantly downregulated the expression of the Kiss1 and Cgrp genes in the hypothalamus. The mechanism may be related to the upregulation of adiponectin secretion and enhanced dopaminergic and GABAergic inhibitory inputs, suggesting that naringin—a flavonoid phytoestrogen widely found in medicinal and dietary substances such as orange peel, Hualujing, and Buddha’s hand—can similarly improve neuroendocrine dysfunction in PMOS by regulating the hypothalamic kisspeptin–GnRH neuronal network.

#### 4.5.2. Clinical Studies

Currently, clinical studies specifically targeting the use of bioactive components from food and medicine with similar properties to improve PMOS through HPO axis regulation remain limited; however, clinical investigations of certain herbal medicines or individual bioactive components have provided preliminary evidence in this regard. In a randomized, double-blind, placebo-controlled trial involving 50 patients with PCOS, Saghafi et al. [99] reported that 12 weeks of treatment with crocin (15 mg/day) in combination with metformin was associated with increases in serum FSH levels, reductions in the Ferriman–Gallwey hirsutism score, and improvement in acne severity, suggesting that crocin may improve PCOS-related hyperandrogenism; however, this small trial (n = 50) with a short intervention period (12 weeks) provides preliminary evidence requiring confirmation in larger randomized controlled trials. Similarly, in a randomized, double-blind, placebo-controlled trial involving 60 patients with PCOS, Sridevi et al. [100] found that an 84-day intervention with the standardized *Asparagus racemosus* root extract CL22209 (100 mg/day) significantly reduced ovarian volume, cyst size, and the number of follicles; it also reduced the LH/FSH ratio and total testosterone, increased SHBG, and improved HOMA-IR. This suggests that *Asparagus racemosus*, as a food–medicine plant, can improve ovarian morphology and endocrine disorders associated with PMOS through the action of its active components, which regulate gonadotropin secretion via the HPO axis and enhance insulin sensitivity. Although the aforementioned clinical studies used plant extracts or single active ingredients as interventions, the source herbs all fall within the category of food–medicine dual-use plants, and their effects all involve the regulation of HPO axis-related hormone levels. This provides preliminary clinical evidence suggesting that active ingredients from MFH plants may improve PMOS through neuroendocrine pathways; however, the overall certainty of evidence remains low due to small sample sizes, short intervention durations, and inconsistent dosing.

**Figure 3:**
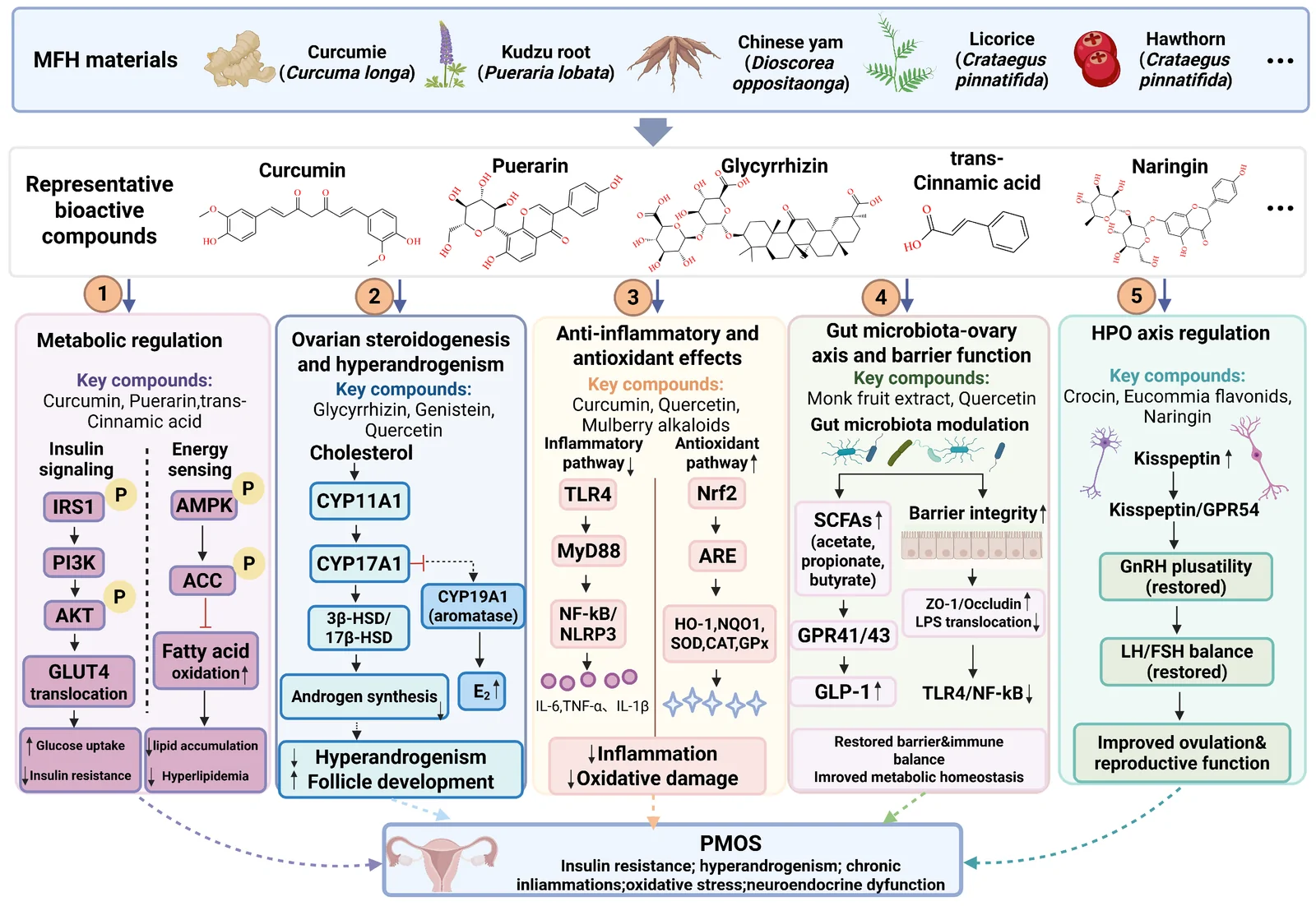
Multi-target mechanisms of MFH-derived bioactive compounds in PMOS. Schematic overview of the mechanisms by which MFH-derived bioactive compounds intervene in PMOS pathophysiology. The diagram illustrates five interconnected pathological domains of PMOS (central column) and their corresponding MFH bioactive compounds (left column) with underlying molecular pathways (right column). These pathways encompass (1) metabolic regulation through insulin signaling (PI3K/AKT/GLUT4) and fatty acid oxidation; (2) ovarian steroidogenesis modulation via CYP11A1/CYP17A1/CYP19A1 to alleviate hyperandrogenism; (3) anti-inflammatory and antioxidant effects through inhibition of TLR4/MyD88/NF-κB/NLRP3 cascades and activation of Nrf2-mediated antioxidant enzymes; (4) gut microbiota–ovary axis regulation via SCFAs/GPR41/43 signaling and intestinal barrier preservation; and (5) neuroendocrine homeostasis through Kisspeptin/GPR54-mediated GnRH pulsatility and LH/FSH balance restoration. Collectively, these multi-target, multi-pathway interventions address the core pathological features of PMOS, including IR, hyperandrogenism, chronic inflammation, OS, and neuroendocrine dysfunction. The arrows indicate the regulatory effects: “↓” denotes inhibition or reduction, and “↑” denotes promotion or increase.

Although these mechanistic findings support the biological plausibility of MFH bioactive compounds in PMOS, most evidence remains preclinical or indirect (Figure 3). Translating these mechanistic insights into functional foods therefore requires formulation strategies, food-grade delivery systems, safety assessment, and product validation, which are addressed in the next section.

**Table 1:** Summary of Preclinical Studies: Mechanism of Action of the Active Ingredient in MFH in the PMOS Model.

Pathophysiology Section	Active Ingredients	Source of Medicinal Materials	Models	Model Relevance	Evidence Level	Administration Method	Pathway and Mechanism of Action	Outcome	Ref.
**IRand glucolipid metabolism**	Curcumin	*Curcuma longa*	DHEA-induced PCOS mouse model	PMOS-specific	Direct evidence	200 mg/kg, oral gavage, 45 d	Suppressed TLR4/MyD88/NF-κB signaling pathway; improved intestinal mucosal barrier via upregulation of ZO-1 and occludin	Insulin sensitivity↑; BW↓; T↓; LH↓; LH/FSH↓; E2↑; cystic follicles↓; corpora lutea↑; plasma LPS↓	[46]
	Curcumin	*Curcuma longa*	Letrozole-induced PCOS rat model	PMOS-specific	Direct evidence	200 mg/kg, i.g., 2 weeks	Modulated IRS1/PI3K/GLUT4 signaling pathway via upregulation of PTEN	FBG↓; FINS↓; HOMA-IR↓; T↓; E2↓; LH↓; P↑; IRS1↓; PI3K↓; AKT↓; GLUT4↑; PTEN↑	[47]
	Puerarin	*Pueraria lobata*	HFFD-induced obese rat model; FFA + fructose-induced HepG2 cells	Other metabolic disorder	Indirect mechanistic evidence	0.2% (*w*/*w*) in diet, 16 weeks (in vivo); 75–150 µM, 24 h (in vitro)	Activated AMPK and PI3K/Akt pathways; upregulated GLUT4; inhibited lipogenic genes via AMPK/ACC	FBG↓; FINS↓; HOMA-IR↓; TG↓; TC↓; p-AMPK↑; p-Akt↑; GLUT4↑	[48]
	Puerarin	*Pueraria lobata*	HG-induced HepG2 cells	Other metabolic disorder	Indirect mechanistic evidence	5–50 µM, 24 h	Activated GPER-mediated calcium signaling; upregulated SIRT1 and AMPK; suppressed SREBP-1c and FASN; enhanced ATGL	TG↓; p-AMPK↑; SIRT1↑; SREBP-1c↓; FASN↓; ATGL↑	[49]
	CYPB	*Dioscorea opposita*	HFD + STZ-induced T2DM mouse model	Other metabolic disorder	Indirect mechanistic evidence	100, 200, 400 mg/kg, i.g., 6 weeks	Activated PI3K/Akt signaling pathway; upregulated InsR, PI3K, Akt, and GLUT4; downregulated FoxO3	FBG↓; PBG↓; OGTT AUC↓; hepatic glycogen↑; GSP↓; InsR↑; PI3K↑; Akt↑; GLUT4↑; FoxO3↓	[50]
	trans-Cinnamic acid	*Cinnamomum cassia*	HFD-induced zebrafish; PA + OA-induced ZFL and HepG2 cells	Other metabolic disorder	Indirect mechanistic evidence	50–100 mg/kg in diet, 14 d (in vivo); 10–50 µM, 24 h (in vitro)	Directly bound and activated AMPKα1; suppressed SREBP1, PPARγ, and FAS; induced lipophagy via AMPK-mTOR axis	Hepatic TG↓; TC↓; LDL-C↓; SREBP1↓; PPARγ↓; FAS↓; p-AMPK↑; LC3B II/I↑; P62↓	[51]
**Regulation of the Local Ovarian Microenvironment and Steroid Synthesis**	Glycyrrhizic acid, liquiritin, genistein, esculetin	Erchen Decoction (ECD): *Pinellia ternata*, *Citrus reticulata*, *Poria cocos*, and *Glycyrrhiza uralensis*	Letrozole + HFD-induced PCOS rat model; DHT-induced KGN cells	PMOS-specific	Direct evidence	4.4 or 8.8 g/kg, i.g., 28 d (in vivo); 50–600 µM, 24 h (in vitro)	Regulated steroid biosynthesis via CYP17A1, HSD11B1, AKR1D1, and HSD11B2	T↓; E2↑; LH/FSH↓; CYP17A1↓; HSD11B1↓; AKR1D1↑; HSD11B2↑	[61]
	Liquiritin	*Glycyrrhiza uralensis*	Letrozole- or DHEA-induced PCOS mouse model	PMOS-specific	Direct evidence	20 mg/kg, i.g., 21 d	Downregulated Cyp17a1, Cyp19a1, Fshr, Runx2, and Ccn2; reduced steroidogenesis and follicle development	TT↓; DHEA-S↓; cystic follicles↓; Cyp17a1↓; Cyp19a1↓; Fshr↓; Runx2↓; Ccn2↓	[62]
	Licorice ethanol extract	*Glycyrrhiza uralensis*	Letrozole-induced PCOS rat model	PMOS-specific	Direct evidence	300 mg/kg, i.g., 2 weeks	Regulated LH/FSH ratio; upregulated Cyp11a1 and Ptgs2; restored follicular development	FSH↑; LH/FSH↓; Cyp11a1↑; Ptgs2↑; follicular cysts↓; antral follicles↑; theca layer thickness↓	[63]
	Soy isoflavones	*Glycine max*	Letrozole-induced PCOS rat model	PMOS-specific	Direct evidence	50 or 100 mg/kg, i.g., 14 d	Inhibited 3β-HSD and 17β-HSD; reduced testosterone; restored follicular development	Testosterone↓; 3β-HSD↓; 17β-HSD↓; ovarian cysts↓; antral follicles↑	[64]
	Quercetin	Quercetin standard	Letrozole-induced PCOS rat model	PMOS-specific	Direct evidence	75 mg/kg, i.g., 28 d	Downregulated CYP11A and CYP17A; upregulated CYP19A; reduced OS	CYP11A↓; CYP17A↓; CYP19A↑; atretic follicles↓; corpora lutea↑	[65]
	Genistein	*Glycine max*	DHEA-induced PCOS mouse model; primary granulosa cells	PMOS-specific	Direct evidence	8.75 mg/kg, i.p., 15 d (in vivo); 100 µM, 24 h (in vitro)	Activated ER-Nrf2-Foxo1-ROS pathway; restored Star, Cyp11a1, Hsd3b1, Cyp17a1, and Cyp19a1 expression	T↓; LH/FSH↓; AMH↓; cystic follicles↓; Star↑; Cyp11a1↑; Hsd3b1↑; Cyp17a1↓; Cyp19a1↓	[66]
	Naringenin, nobiletin	*Pinellia ternata*, *Citrus reticulata*	HFD + letrozole-induced PCOS rat model; DHT-induced KGN cells	PMOS-specific	Direct evidence	1.8 g/kg, i.g., 28 d (in vivo); 100 µM each, 24 h (in vitro)	Regulated steroid biosynthesis via CYP17A1, CYP19A1, AKR1C3, and HSD17B1	T↓; E2↑; cystic follicles↓; corpora lutea↑; CYP17A1↓; CYP19A1↓; AKR1C3↑; HSD17B1↑	[67]
**Anti-inflammatory Effects and Alleviation of OS**	Curcumin	*Curcuma longa*	DHEA-induced PCOS rat model	PMOS-specific	Direct evidence	50 mg/kg, i.g., 21 d	Upregulated PPAR-γ; reduced ROS and MDA; increased SOD, GPx, and GSH	ROS↓; MDA↓; SOD↑; GPx↑; GSH↑; PPAR-γ↑; IR↓; T↓; LH/FSH↓	[75]
	Curcumin	*Curcuma longa*	DHEA-induced PCOS mouse model	PMOS-specific	Direct evidence	200 mg/kg, i.g., 45 d	Suppressed TLR4/MyD88/NF-κB signaling; restored intestinal barrier via ZO-1 and occludin	T↓; LH/FSH↓; plasma LPS↓; IL-6↓; TNF-α↓; IL-10↑; ZO-1↑; occludin↑	[46]
	Quercetin	Flavonoid from various plants (e.g., Hawthorn, *Sophora japonica*)	DHEA-induced PCOS rat model	PMOS-specific	Direct evidence	15 mg/kg, i.g., 30 d	Reduced OS via SOD, CAT, GPx, GR, and GSH; downregulated DNMT1 and DNMT3a	SOD↑; CAT↑; GPx↑; GR↑; GSH↑; MDA↓; DNMT1↓; DNMT3a↓; serum Zn↑	[76]
	Quercetin	*Crataegus* spp. (hawthorn)	DHEA-induced PCOS rat model	PMOS-specific	Direct evidence	100 mg/kg, i.g., 28 d	Inhibited TLR4/NF-κB signaling; downregulated p22phox, OX-LDL, and TLR-4; reduced IL-1β, IL-6, TNF-α	HOMA-IR↓; IL-6↓; TNF-α↓; TLR-4↓; NF-κB nuclear translocation↓	[77]
**Regulation of the Gut–Ovarian Axis**	Mogroside-rich extract	*Siraitia grosvenorii*	Letrozole-induced PCOS rat model	PMOS-specific	Direct evidence	600 mg/kg, i.g., 21 d	Modulated gut microbiota via enriching Akkermansia and Parasutterella, reducing Corynebacterium; restored butyric acid and gamma-tocotrienol; suppressed NF-κB/NLRP3 signaling pathway	T↓; HOMA-IR↓; cystic follicles↓; corpora lutea↑; IL-6↓; TNF-α↓; p-NF-κB↓; NLRP3↓; Akkermansia↑; butyric acid↑	[88]
	Quercetin	*Crataegus* spp. (hawthorn)	Letrozole + HFD-induced PCOS mouse model	PMOS-specific	Direct evidence	125 mg/kg, i.g., 45 d	Enriched Akkermansia; promoted SCFAs; enhanced ZO-1 and occludin; inhibited TGF-β/SMAD3 signaling	T↓; LH/FSH↓; insulin sensitivity↑; cystic follicles↓; ZO-1↑; occludin↑; Akkermansia↑; acetate↑; TGF-β↓; α-SMA↓	[89]
	Zishen Qingre Lishi Huayu Recipe	TCM compound	DHEA-induced PCOS mouse model	PMOS-specific	Direct evidence	Fecal microbiota transplantation, oral gavage, 3 weeks	Regulated gut–ovary axis via modulating gut microbiota and butyrate metabolism	T↓; LH↓; E2↓; FSH↑; cystic follicles↓; butyric acid↑; Lachnospira↑; Faecalibacterium↑; Escherichia-Shigella↓	[90]
**Regulation of the HPO Axis and Neuroendocrine Homeostasis**	Crocetin	*Crocus sativus* (saffron)	Prenatal DHT-induced PCOS mouse model	PMOS-specific	Direct evidence	40 mg/kg, i.g., 4 weeks	Regulated HPO axis via modulating Kisspeptin neurons; restored GnRH and LH/FSH ratio	LH/FSH↓; T↓; E2↑; estrous cycle normalized; cystic follicles↓; AVPV kisspeptin↑; ARC kisspeptin↓	[96]
	TFEL	*Eucommia ulmoides* Oliv. leaves	Letrozole + HFD-induced PCOS-IR rat model	PMOS-specific	Direct evidence	55, 110, 220 mg/kg, i.g., 21 d	Regulated HPO axis via modulating Kiss1/IGF-1/LEPR/AR; activated ovarian PI3K/AKT signaling	BW↓; ovarian index↓; E2↑; T↓; HOMA-IR↓; Kiss1 (hypothalamus)↓; Kiss1 (pituitary)↑; Kiss1 (ovary)↑; p-AKT↑; GLUT4↑	[97]
	Naringenin	*Citrus* spp. (citrus fruits)	Estradiol valerate-induced PCOS rat model	PMOS-specific	Direct evidence	20 or 50 mg/kg, i.p., 14 d	Downregulated hypothalamic Kiss1 and Cgrp gene expression	Kiss1↓; Cgrp↓	[98]

**Abbreviations:** AMH, anti-müllerian hormone; AR, androgen receptor; ATGL; adipose triglyceride lipase; AVPV, anteroventral periventricular nucleus; BW, Body weight; CAT, catalase; Cgrp, calcitonin gene-related peptide; CYP11A1, Cytochrome P450 family 11 subfamily A member 1; DHEA, dehydroepiandrosterone; DHEA-S, dehydroepiandrosterone sulfate; DHT, dihydrotestosterone; DNMT, DNA methyltransferase; FAS, fatty acid synthase; FASN, fatty acid synthase; FBG, Fasting blood glucose; FINS, fasting insulin; Fshr, follicle-stimulating hormone receptor; GLUT4, glucose transporter 4; GnRH, gonadotropin-releasing hormone; GPx, glutathione peroxidase; GSP, glycated serum protein; HPO axis, hypothalamus–pituitary–ovary axis; HSD11B1, 11β-hydroxysteroid dehydrogenase 1; IGF-1, insulin-like growth factor 1; IRS-1, insulin receptor substrate 1; LEPR, Leptin receptor; MDA; malondialdehyde; OGTT, oral glucose tolerance test; PTEN, phosphatase and tensin homolog; SHBG, Sex hormone-binding globulin; SOD, superoxide dismutase; SREBP-1c, sterol regulatory element-binding protein 1c; T2DM, type 2 diabetes mellitus; TFEL, total flavonoids from *Eucommia ulmoides* Oliv. Leaves; TGF-β, transforming growth factor-beta; TLR-4, toll-like receptor 4; PMOS, polyendocrine metabolic ovarian syndrome; MFH, medicine-food homology; PMOS-specific, models directly relevant to PMOS pathophysiology, including letrozole-, DHEA-, DHT-, estradiol valerate-, or clomiphene-induced rodent models, PMOS-related cellular models (e.g., KGN cells and primary granulosa cells), and PMOS patient-derived samples; Other metabolic disorder, non-PMOS metabolic models, including high-fat diet-induced obesity, streptozotocin-induced type 2 diabetes, HepG2 hepatic steatosis, and high-fat diet-fed zebrafish, which provide only indirect mechanistic evidence. ↑ indicates an increase or upregulation; ↓ indicates a decrease or downregulation.

**Table 2:** Clinical Applications of MFH Active Ingredients in PMOS.

Pathophysiology Section	Active Ingredients	Source of Medicinal Materials	Sample Size	Gender/Age	Treatment	Outcome	Ref.
IR **and glucolipid metabolism**	Curcumin	*Curcuma longa*	104 (97 completed)	Female/18–45 yr	500 mg/bid plus DASH diet or standard diet, oral, 12 weeks	Insulin↓; HOMA-IR↓	[52]
	Curcumin	*Curcuma longa*	3 RCTs (85 intervention/83 placebo)	Female/adults	500 mg/d–1500 mg/d, oral, 6–12 weeks	FPG↓; FI↓; HOMA-IR↓; TC↓	[53]
	Cinnamon	*Cinnamomum* spp.	7 RCTs (373/310 for FBS/HOMA-IR)	Female/adults with PCOS	500–1500 mg/d, oral, 6–52 weeks	FBS↓; HOMA-IR↓; TC↓; LDL↓; Weight↓	[54]
	Licorice crude extract	*Glycyrrhiza glabra* L.	75 (25 Diane-35 + licorice/25 Diane-35 alone/25 healthy controls)	Female/20–40 yr	450 mg/bid crude licorice extract capsules plus Diane-35, oral, 12 weeks	Insulin↓; HOMA-IR↓; FBS↓; TC↓; HDL↑; IL-1β↓; TAC↑	[55]
**Regulation of the Local Ovarian Microenvironment and Steroid Synthesis**	Licorice extract	*Glycyrrhiza glabra* L.	66 (33 licorice + LCD/33 placebo + LCD)	Female/18–45 yr, overweight/obese (BMI 25–35 kg/m^2^)	1500 mg/d (3 × 500 mg capsules) plus low-calorie diet (1200–1800 kcal/d), oral, 8 weeks	FSH↑; LH/FSH ratio↓; Testosterone↓	[68]
	Marjoram (*Origanum majorana*) + Cinnamon (*Cinnamomum zeylanicum*) herbal tea	*Origanum majorana* L. (Lamiaceae) + *Cinnamomum zeylanicum* Blume (Lauraceae)	36 completed (21 herbal/15 placebo)	Female/16–36 yr	Herbal tea, oral, 12 weeks	FSH↑; AMH↓	[69]
**Anti-inflammatory Effects and Alleviation of OS**	curcumin capsules or water decoction	*Curcuma longa* L.	7 RCTs (447 total: 225 intervention/222 control)	Female/adults with PCOS	80–1500 mg/d curcumin, oral, 6 weeks–6 months	CRP↓; FBG↓; INS↓; HOMA-IR↓; QUICKI↑; TC↓	[78]
	Sangzhi alkaloids	*Morus alba* L.	128 (88 SZ-A/40 placebo)	Female/18–45 yr	300 mg/d, oral, 24 weeks	FAI↓; SHBG↑; BMI↓; Waist-to-height ratio↓; Insulin sensitivity improved	[81]
**Regulation of the Gut–Ovarian Axis**	ZQLHR	Traditional Chinese medicinal compound (including *Glycyrrhiza uralensis*, *Prunus persica*)	25	Female/18–35 yr	ZQLHR water decoction, oral, 6 months	Total T↓; LH↓; LH/FSH ratio↓; 2h INS↓; Lachnospira↑; Faecalibacterium↑; Escherichia-Shigella↓; butyrate metabolism↑	[90]
	WTP diet ± Acarbose	WTP diet (high-fiber diet containing whole grains, medicinal foods, and prebiotics)	25 (14 W group/11 A group)	Female/15–41 yr	WTP diet ± acarbose 50 mg tid, 12 weeks	A group: LH/FSH ratio↓; Testosterone↓; HOMA-IR↓; Bifidobacterium-enriched CAGs↑; B. vulgatus-dominated CAGs↓	[91]
**Regulation of the HPO Axis and Neuroendocrine Homeostasis**	Crocin	*Crocus sativus* L. (saffron)	50 (25 crocin + metformin/25 placebo + metformin)	Female/18–44 yr	Crocin 15 mg/qd + metformin 500 mg/bid, oral, 12 weeks	FSH↑; Ferriman–Gallwey score↓; Acne severity↓	[99]
	CL22209	*Asparagus racemosus* Willd. (Shatavari)	60 (30 CL22209/30 placebo)	Female/20–35 yr	CL22209 100 mg/qd, oral, 84 days	Ovarian volume↓; Follicle number↓; LH/FSH ratio↓; FSH↑; SHBG↑; Testosterone↓; HOMA-IR↓; Menstrual interval↓; mFG score↓	[100]

**Abbreviations:** DASH, Dietary Approaches to Stop Hypertension; HOMA-IR, homeostatic model assessment of insulin resistance; FPG, fasting plasma glucose; FI, fasting insulin; TC, total cholesterol; LDL, low-density lipoprotein cholesterol; HDL, high-density lipoprotein cholesterol; TAC, total antioxidant capacity; CAGs, co-abundant groups; mFG, modified Ferriman–Gallwey; SHBG, sex hormone-binding globulin. ↑ indicates an increase or upregulation; ↓ indicates a decrease or downregulation.

## 5. Advanced Extraction, Encapsulation, and Stabilization Technologies

The efficient utilization of MFH’s active ingredients faces three major technical bottlenecks: extraction efficiency, oral bioavailability, and formulation stability. Addressing the inherent limitations of polyphenols and flavonoids—such as curcumin and quercetin—including their high heat sensitivity, poor water solubility, low gastrointestinal stability, and significant first-pass metabolism, the synergistic development of green extraction technologies, nano-encapsulation technologies, and controlled-release delivery systems in recent years has provided viable technical pathways for their application in functional foods and precision nutrition interventions. Given that these technologies are intended for functional food applications, their food-grade suitability requires critical assessment [101]. Accordingly, the evaluation of extraction, encapsulation, and stabilization technologies in this section considers not only technical performance but also their compatibility with food-grade requirements [102]. The evaluation of extraction, encapsulation, and stabilization technologies in this section considers not only technical performance but also their compatibility with food-grade requirements [102,103]. Three criteria are applied: (1) material safety, i.e., whether the carrier materials and solvents are food-grade or GRAS-certified; (2) scalability, i.e., whether the technology has been validated or can be adapted for industrial-scale food production; (3) Food matrix compatibility, i.e., whether the technology preserves sensory quality and complies with the regulatory framework for food [104]. Technologies developed primarily for pharmaceutical applications are discussed when they provide insights into mechanisms of action, but their suitability for food-grade use is explicitly evaluated [105]. A comprehensive summary of the extraction, encapsulation, and stabilization technologies discussed in this section is provided in Table 3.

### 5.1. Green and High-Efficiency Extraction Technology

Active components in MFH plants—polyphenols, flavonoids, and isoflavonoids (e.g., curcumin, hawthorn flavonoids, quercetin, and puerarin)—are heat-sensitive and chemically unstable; conventional thermal reflux and organic solvent extraction often cause oxidative degradation and bioactivity loss. Green extraction technologies aim to achieve efficient recovery under mild conditions while ensuring environmental sustainability [106]. Ultrasonic-assisted extraction (UAE) disrupts plant cell walls through localized high pressure and shear forces generated by the cavitation effect, significantly improving mass transfer efficiency at lower temperatures [107]. Sadeghi-Kiakhani et al. used UAE to extract pigments from hawthorn berries and optimized the extraction parameters using response surface methodology (RSM). They found that the optimal extraction efficiency was achieved by treating the sample for 40 min at 60 °C in an ethanol–water mixed solvent system. Major flavonoid components, including quercetin, rutin, and kaempferol, were identified using FTIR and HPLC techniques [108]. Enzyme-assisted extraction can further break down the polysaccharide framework of plant cell walls, thereby enhancing the release efficiency of bioactive compounds. Studies have shown that the synergistic treatment of hawthorn fruits with cellulase and pectinase significantly increases total phenolic content and antioxidant activity, with vanillic acid and ferulic acid levels increasing by 115.6-fold and 93.9-fold, respectively [109]. Microwave-assisted extraction (MAE) uses dielectric heating to shorten extraction time, suitable for curcumin. Supercritical CO_2_ extraction (SFE), mild and solvent-free (31.1 °C, 7.38 MPa), suits isoflavones like puerarin; Wang et al. achieved 5.8 ± 0.3 mg/g puerarin at 50 °C, 20 MPa [106,110].

In recent years, natural eutectic solvents (NADES) have attracted widespread attention in the field of plant bioactive compound extraction as a new class of biodegradable, low-toxicity green solvents. Thanks to their adjustable polarity and ability to form strong hydrogen bonds, NADES exhibit excellent solubility for both lipophilic and hydrophilic bioactive compounds, such as curcumin [111]. Studies have shown that combining NADES with enhanced extraction techniques, such as MAE or UAE, can synergistically improve the extraction efficiency and antioxidant activity of polyphenols and flavonoids [112]. For heat-sensitive compounds, optimization should control temperature (<70 °C), solvent composition (e.g., ethanol–water ratio), time, and solid-to-liquid ratio to balance extraction efficiency and stability. These green extraction technologies provide the basis for high-quality preparation of MFH bioactives and their large-scale application in PMOS functional foods. Among the green extraction technologies mentioned above, UAE, MAE, enzyme-assisted extraction, and SFE all meet food-grade requirements and have already been applied on a large scale in the food industry; although NADES can be formulated as a food-grade solvent, its industrial scalability still requires further validation.

### 5.2. Bioavailability Enhancement Technologies

Most active ingredients in MFH are polyphenols or flavonoids, and their oral bioavailability is limited by poor water solubility, enzymatic degradation in the gastrointestinal tract, low intestinal epithelial permeability, and significant first-pass metabolism [113]. Nanoencapsulation offers an effective solution. Lipid-based systems (liposomes, nanoemulsions, SLNs) encapsulate hydrophilic and lipophilic components, enabling controlled release and improved solubility. Polymer nanoparticles using biodegradable polymers such as chitosan prolong circulation by optimizing size and surface properties. Raja et al. prepared Arg-CS-NAcHis/Cur nanoparticles (~200 nm, +39.8 mV) with sustained release and superior cellular activity versus free curcumin. In an estradiol valerate-induced PMOS rat model, these nanoparticles improved serum hormones and ovarian morphology, supporting their potential as curcumin delivery carriers for PMOS [114].

Targeting the characteristics of OS in the ovarian microenvironment associated with PCOS, Li et al. developed a macrophage-membrane-mimicking, reactive oxygen species (ROS)-responsive quercetin nanodelivery system (MM@PCD@QNPs), with a particle size of approximately 133.63 nm. This system achieves immune evasion and active targeting of ovarian inflammatory lesions via the CD11b and CD47-SIRPα proteins on the macrophage membrane surface. In a DHEA-induced PCOS mouse model, it promoted granulosa cell proliferation, inhibited apoptosis, and reduced ROS levels by activating the MAPK7-Nrf2-NQO1 signaling pathway, thereby significantly improving ovarian cystic morphology, sex hormone dysregulation, and fertility [115]. Additionally, a combined nanopreparation of genistein and daidzein (198–450 nm, encapsulation efficiency > 90%) improved IR and OS by upregulating the SIRT1/PGC-1α pathway in a letrozole-induced PMOS rat model, restoring hormonal homeostasis and follicular structure. Its efficacy was superior to that of the parent drug and comparable to clomiphene, confirming that the nanodelivery strategy can significantly enhance the anti-PCOS bioactivity of isoflavone phytoestrogens [116].

Self-microemulsifying drug delivery systems (SMEDDS) are isotropic mixtures composed of an oil phase, an emulsifier, and a co-emulsifier that spontaneously form oil-in-water microemulsions with particle sizes less than 100 nm under the influence of gastrointestinal peristalsis. By increasing the contact area between the drug and the intestinal epithelium, protecting the active ingredients from enzymatic degradation, and promoting lymphatic transport to bypass first-pass metabolism, this system significantly enhances the oral bioavailability of poorly soluble MFH active ingredients [117]. For most MFH polyphenol/flavonoid components, such as curcumin, quercetin, and puerarin, oral bioavailability is generally limited by poor water solubility and gastrointestinal instability. SMEDDS can increase the solubility of curcumin by more than 100-fold and boost the small intestinal absorption rate constant by more than 2-fold. The curcumin–sorafenib self-emulsifying microemulsion delivery system prepared by Huang et al. increased the oral bioavailability of curcumin by 3.64-fold, and the combination of the two exhibited a significant synergistic antitumor effect (CI = 0.55), confirming that SMEDDS can effectively enhance the oral absorption efficiency of poorly soluble active ingredients and their potential for combination therapy [118]. This strategy overcomes the inherent limitations of natural active ingredients through pharmaceutical formulation techniques, providing a viable delivery solution for the transformation of MFH-derived compounds into functional foods. By improving pharmacokinetic properties and targeting capabilities, the aforementioned nanodelivery strategies offer new approaches for precision nutritional interventions in PMOS.

The aforementioned lipid-based systems and biodegradable polymer nanoparticles, such as those made from chitosan, can typically be prepared using food-grade or GRAS-certified materials and are well-suited for food applications [101]. While SMEDDS can effectively improve the oral bioavailability of ingredients such as curcumin, they often require the synthesis of high-concentration surfactants and solubilizers, which may raise sensory and regulatory concerns in food applications. Furthermore, the food-grade suitability of complex delivery systems, such as those mimicking macrophage membranes, still requires further evaluation due to the special materials and processes involved [119].

### 5.3. Stability and Controlled-Release Technology

Polyphenolic MFH active ingredients (such as curcumin, anthocyanins, and quercetin) are highly sensitive to light, heat, pH, and oxygen; they are prone to oxidative degradation or isomerization during processing and storage, leading to a significant loss of bioactivity [120]. Microencapsulation technology effectively isolates active ingredients from adverse environmental factors by embedding them within polysaccharide (e.g., alginate, chitosan, maltodextrin) or protein matrix materials to form micron-scale core-shell structures [121]. Spray drying is the main industrial method for heat-sensitive substances due to simplicity and cost control. Martinić et al. first microencapsulated dandelion leaf extract by spray drying (130 °C); pectin achieved the highest encapsulation efficiency (74.4%) for chicoric acid, while alginate provided the most sustained release. This confirmed that the selection of wall materials can simultaneously achieve the protection and controlled release of heat-sensitive polyphenols, providing a formulation strategy for the functional food transformation of MFH’s active ingredients [122].

Cyclodextrin inclusion protects MFH actives from environmental degradation. Novel γ-cyclodextrin metal-organic frameworks (γ-CD-MOFs) encapsulated quercetin, apigenin, and curcumin with efficiencies of 82.22%, 70.30%, and 64.10%, increasing solubility (222-, 322-, 101-fold) and bioavailability (18-, 19-, 145-fold), with good photochemical/thermal stability and controlled release [123]. However, although the aforementioned cyclodextrin-based delivery systems can effectively improve the physicochemical stability and absorption characteristics of active ingredients, they struggle to achieve active targeting of the lesion site and spatiotemporally controlled release. To address this limitation, enzyme-responsive carriers based on natural polysaccharides have emerged; these systems utilize enzymes specific to the colonic microbiota (such as glycosidases) to degrade the polysaccharide backbone, triggering the localized release of the active ingredient in the colon and thereby achieving precise colonic drug delivery [124]. Gut microbiota dysbiosis and colonic microenvironment changes are critical in PMOS progression (Section 4.4). This strategy can precisely deliver MFH actives to the microbiota–ovarian axis interface, enhancing targeted intervention in PMOS. These technologies provide a pharmaceutical basis for stabilizing MFH actives in PMOS functional foods.

In stabilization and controlled-release applications, spray drying and composite agglomeration have been widely adopted in the food industry, relying on relatively inexpensive equipment and food-grade binders such as maltodextrin, gum arabic, and gelatin [125]. Cyclodextrin inclusion, particularly γ-cyclodextrin, also meets food-grade requirements, as cyclodextrins are approved food additives [126]. However, while nano-γ-CD-MOFs offer high encapsulation efficiency and enhanced bioavailability, their synthesis involves metal–organic frameworks, which may raise additional regulatory concerns regarding metal residues and long-term safety in food applications [127]. Enzyme-responsive, colon-specific carriers based on natural polysaccharides show conceptual promise for food applications, but their scalability and long-term stability in food matrices still require further validation.

**Table 3:** Core Technical Strategies for the Efficient Extraction, Delivery, and Stabilization of Bioactive Compounds from Plant Sources.

Technology Category	Technology/Systems	Core Principles	Primary Target Audience	Main Effects	Ref.
**Green and High-Efficiency Extraction Technology**	UAE	Cavitation-induced cell wall disruption enhances mass transfer at mild temperatures.	Hawthorn (*Crataegus elbursensis*) flavonoids (quercetin, rutin, kaempferol).	UAE improved efficiency 20–70% vs. conventional; RSM-optimized at 8 g/L, pH 5, 60 °C, 40 min in ethanol–water (W1/4E); rutin most abundant	[108]
	Enzyme-Assisted Extraction	Carbohydrate-cleaving enzymes hydrolyze glycosidic bonds between phenolics and cell wall polymers, releasing free phenolic aglycones.	Hawthorn (*Crataegus orientalis*) fruits; target phenolics including vanillic acid and ferulic acid.	Enzyme synergy enhanced TPC from 885 to 1899 mg GAE/100g DM; vanillic acid and ferulic acid increased.	[109]
	SFE	CO_2_ above critical point offers gas-like diffusivity and liquid-like solvating power for nonpolar compounds.	Puerarin (isoflavones) from *Pueraria* spp.; target compounds: isoflavones, puerarin.	CO_2_ mild critical conditions enable solvent-free extracts; optimized at 50 °C, 20 MPa yielded 5.8 ± 0.3 mg/g puerarin.	[106,110]
	MUAE + NADES	Microwave-induced cell disruption and ultrasound cavitation synergistically enhance mass transfer; NADES provides tunable polarity.	Curcumin from *Curcuma longa* rhizomes; target compound: curcumin.	MUAE + NADES achieved 40.72 mg/g curcumin, 14.36% higher than UAE, with 50% less solvent at 20% water content, 8% solid loading.	[112]
**Bioavailability Enhancement Technologies**	Arginine and N-Acetyl Histidine-Modified Chitosan Self-Assembled Nanoparticles	Amphiphilic chitosan self-assembles into nanoparticles enabling sustained curcumin release.	Curcumin	NPs (200 nm) showed sustained release, superior uptake, and reversed PCOS in rats.	[114]
	Macrophage Membrane-Cloaked ROS-Responsive Quercetin Nanocarriers	Macrophage membrane coating enables immune evasion via CD11b/CD47-SIRPα; PBAP confers ROS-triggered drug release.	Quercetin	NPs (133.63 nm): ROS-release, lesion targeting, MAPK7-Nrf2-NQO1 activation, PCOS/fertility improvement.	[115]
	Daidzein and Genistein Polymeric Nanoparticles	Polymeric nanoparticles enhance solubility and oral bioavailability of poorly soluble isoflavones.	Daidzein; Genistein	NPs activated SIRT1/PGC-1α, restored hormones/follicles, comparable to clomiphene.	[116]
	SMEDDS for Sorafenib-Curcumin Co-Administration	SMEDDS forms oil-in-water microemulsions (<100 nm), enhancing solubility and lymphatic absorption of lipophilic drugs.	Curcumin; Sorafenib	SMEDDS improved curcumin bioavailability 3.64-fold and exhibited synergistic anti-tumor effect.	[118]
**Stability and Controlled-Release Technology**	**Spray Drying Microencapsulation**	Spray drying encapsulates sensitive polyphenols in biopolymer carriers to protect against degradation.	Chicoric acid	Pectin achieved 74.4% chicoric acid EE; alginate provided the slowest sustained release in simulated GI conditions.	[122]
	**nano-γ-CD-MOFs**	γ-CD-MOFs provide a hydrophilic porous framework with hydrophobic cavities for encapsulating insoluble polyphenols via impregnation.	Quercetin; Apigenin; Curcumin	Nano-γ-CD-MOFs achieved EE of 82.22%/70.30%/64.10%, solubility ↑222/322/101×, bioavailability ↑18/19/145×, with sustained release.	[123]

**Abbreviations**: SFE, Supercritical Fluid Extraction; MUAE, Microwave-Ultrasound-Assisted Extraction; NADES, Natural Deep Eutectic Solvent; SMEDDS, Self-Microemulsifying Drug Delivery System; nano-γ-CD-MOFs, Nano-γ-Cyclodextrin Metal–Organic Frameworks. ↑ indicates an increase or upregulation.

## 6. Functional Food Development and Innovative Applications

Building on these formulation technologies, this section addresses their translation into functional food products. The growing body of preclinical and clinical evidence has laid the foundation for the development of functional foods containing MFH active ingredients. Given that lifestyle interventions are the first-line strategy for patients with PMOS, product forms such as meal replacement powders, functional teas, fermented beverages, and dietary supplements provide highly accessible and well-tolerated vehicles for daily intake. This section explores the relationship between functional food development, the pathophysiological mechanisms of PMOS, and the active components of MFH from the perspectives of product formats and formulation design. It distinguishes between two types of evidence: formulations that have been evaluated in clinical studies, and conceptual proposals based on mechanisms, network pharmacology, or preclinical evidence but have not yet undergone product-level validation. The evidence status of each formulation is clearly labeled, and proposals that have not undergone product-level validation are listed as areas requiring future validation.

### 6.1. Meal Replacement Powders

Weight management is central to lifestyle interventions for PMOS, and guidelines recommend weight loss of 5–10%. While meal replacement powders can aid in weight loss, they cannot address the multifaceted pathophysiological mechanisms involved. Incorporating MFH components with insulin-sensitizing, anti-inflammatory, and antioxidant properties holds promise for achieving multidimensional metabolic benefits beyond weight loss alone.

Based on the above design rationale, the following MFH active ingredients may be considered for incorporation into meal replacement powders: Curcumin: systematic reviews and meta-analyses have confirmed that curcumin supplementation was associated with significant reductions in fasting blood glucose, fasting insulin, and HOMA-IR in women with PCOS [128]; Green tea catechins: randomized controlled trials have confirmed that daily green tea supplementation significantly reduces body weight, BMI, waist circumference, and body fat percentage in women with PCOS; moreover, the reduction in IL-6 levels is significantly correlated with improvements in these indicators, suggesting that it may indirectly modulate the inflammatory state through weight loss [129]; Cinnamon polyphenols: a systematic review and meta-analysis showed that cinnamon supplementation marginally improves HOMA-IR in women with PCOS; however, the certainty of the evidence is low, and further validation is needed [130]. These three components correspond to the three core dimensions of the PCOS pathophysiological network—energy metabolism (body weight), insulin signaling (IR), and inflammation/OS—forming a complementary metabolic regulation network that builds upon the weight-loss function of the meal replacement powder. However, their incorporation into a combined meal replacement powder has not been clinically evaluated. The following formulation design is therefore a proposal based on mechanistic complementarity and existing supplement evidence, and requires future product-level validation. Formulation design must take into account the bioavailability of active ingredients—fat-soluble components (such as curcumin) can be supplemented with self-microemulsion delivery systems or absorption enhancers such as piperine to ensure effective exposure levels are achieved following oral administration.

### 6.2. Functional Tea Beverages

Functional tea beverages are an accessible product format that integrates MFH’s active ingredients into daily hydration routines, making them particularly suitable for PMOS patients who require long-term lifestyle interventions. Dietary supplements can improve glucose metabolism in PMOS through various mechanisms, such as enhancing insulin sensitivity and increasing anti-inflammatory and antioxidant activity. When using tea beverages as a delivery vehicle for these ingredients, the issue of polyphenol stability during hot water infusion must be addressed [11,131].

Clinical studies have also confirmed that a cinnamon-and-marjoram tea blend can improve FSH and AMH levels in women with PCOS (Majeed et al., 2025), providing preliminary evidence for the development of tea-based products [69]. A systematic review and meta-analysis incorporating six randomized controlled trials (involving a total of 235 women with PCOS) indicated that herbal tea supplementation significantly reduced body weight, body mass index, and fasting blood glucose levels in women with PCOS, while significantly increasing FSH levels. This suggests that herbal teas formulated with green tea, marjoram, and peppermint, which are classified as “food–medicine” plants, can play a supportive role in managing PCOS through multidimensional pathways, including improved weight management, glucose metabolism, and gonadotropin secretion, thereby providing evidence-based medical support for the product development of functional tea beverages as carriers of MFH active ingredients [132]. Furthermore, an updated systematic review, which included 10 randomized controlled trials (involving a total of 548 women with PCOS), found that herbs such as marjoram, green tea, cinnamon, and black cumin—all of which are food–medicine dual-use herbs—can significantly alleviate PCOS-related clinical symptoms through mechanisms such as regulating sex hormone levels, improving IR, and reducing OS, thereby providing evidence-based support for the use of herbal products, such as functional teas, as carriers of active MFH ingredients in adjunctive intervention strategies [133]. To date, the specific tea formulations proposed here have not been independently evaluated as finished products; they are presented as formulation concepts for future development.

### 6.3. Fermented Beverages

Patients with PMOS exhibit gut microbiota dysbiosis and a compromised intestinal barrier; the absorption and metabolism of MFH’s active ingredients are highly dependent on the integrity of the gut microbiome. Fermented beverages provide probiotics and prebiotics that can specifically regulate the gut microbiome and form a synergistic network with MFH components, offering an indirect intervention pathway via the gut–ovarian axis for PMOS management.

When plants classified as “food–medicine” (such as hawthorn, kudzu root, mulberry leaves, and goji berries) are used as fermentation substrates and fermented by probiotics, the release patterns and biotransformation efficiency of their active ingredients can undergo significant changes. Related mechanistic studies support this: Du et al. [134], through a systematic review of multidisciplinary evidence on PCOS-related microbiota, revealed that gut dysbiosis drives IR and ovarian dysfunction via LPS translocation and disruptions in SCFAs, bile acids, and tryptophan metabolites to drive IR and ovarian dysfunction, whereas microbiota-targeted intervention strategies can improve reproductive endocrinology by restoring microbial diversity and metabolite homeostasis. This suggests that microbiota-mediated metabolite conversion (such as the production of estro-mone) may serve as a key fermentation regulation target for enhancing the endocrine-regulatory efficacy of MFH’s active ingredients. A short-term isoflavone intervention study by Haudum et al. [135] in women with PCOS further demonstrated that after soy aglycones were converted to equimaphenol by the gut microbiota, its metabolites were associated with reduced levels of androgens and AMH, accompanied by improved insulin sensitivity and restoration of gut microbiota α-diversity, suggesting that microbiome-mediated isoflavone biotransformation, by enhancing estrogen receptor modulatory activity and antioxidant efficacy, provides preliminary clinical evidence at the mechanistic level for fermentation-based enhancement strategies of food–medicine active ingredients. Furthermore, a systematic review by Yang et al. [8] on patients with PCOS and IR indicated that the combination of probiotics and metformin can synergistically reshape the gut microbiota, increase short-chain fatty acid production, and strengthen the intestinal barrier. This, in turn, enhances AMPK pathway activation and GLP-1 secretion, thereby improving metabolic and hormonal disorders while alleviating metformin-induced gastrointestinal intolerance, further suggesting that fermentation strategies targeting the gut microbiota can serve as a key regulatory pathway for enhancing the efficacy and reducing the toxicity of bioactive components derived from food–medicine dual-use sources.

In summary, the evidence presented above provides a scientific basis for the development of fermented beverages using MFH materials such as hawthorn, kudzu root, and soybeans as substrates. The fermentation process not only releases bound polyphenols and flavonoids from the plant substrates but also enhances their bioavailability through metabolic conversion by probiotics, thereby creating functional fermented products that target PMOS gut–ovarian axis dysfunction. Nonetheless, the fermented beverage formulations proposed here remain conceptual and have not undergone product-level evaluation; future studies should assess their stability, sensory properties, and clinical efficacy.

### 6.4. Dietary Supplements

Dietary supplements represent the most direct product form for translating the active ingredients of MFH from basic research to clinical applications for PMOS. While a substantial body of meta-analysis evidence has been accumulated regarding their role in adjunctive interventions, there are significant differences in the effects of various supplements on different pathological mechanisms, and single-component strategies struggle to comprehensively address the multidimensional metabolic disorders associated with PMOS.

Zhao et al. [136] conducted a network meta-analysis incorporating 79 RCTs involving 5501 participants to systematically evaluate the differential efficacy of various MFH supplements: curcumin was most effective in improving both low-density and high-density lipoprotein cholesterol levels; soy isoflavones were most effective at reducing malondialdehyde and increasing glutathione; inositol significantly reduced total cholesterol and triglycerides; and omega-3 ranked first in improving IR. However, none of the above supplements had a significant effect on total testosterone, SHBG, or dehydroepiandrosterone sulfate, suggesting that while single-component MFH supplements have clear differential value in regulating glucose and lipid metabolism and OS, they are unable to effectively address hyperandrogenism, a core pathological mechanism. A meta-analysis by Wang et al. [137] further confirmed that antioxidant dietary supplements (including phenolic compounds from MFH sources such as curcumin and quercetin, as well as minerals) can significantly improve IR and fasting blood glucose in women with PCOS, but have limited effects on lipid profiles (except for triglycerides). This suggests that the insulin-sensitizing value of MFH’s antioxidant components is prominent, but its metabolic regulatory effects remain selective.

The above evidence suggests that, for patients with PMOS characterized primarily by hyperandrogenism, the development of dietary supplements should prioritize a compound formulation strategy. This involves the rational combination of MFH components that target different pathological pathways, such as cinnamon polyphenols to improve IR, licorice flavonoids to regulate steroid synthesis, and curcumin for its antioxidant effects, to achieve a multi-pathway, synergistic intervention in the hyperandrogenic microenvironment. It should be emphasized that this compound supplement strategy is a rational design based on complementary mechanisms and has not been clinically tested as a fixed-dose combination.

### 6.5. Design of Synergistic Effects in Compound Formulations

The complexity of the pathophysiological mechanisms underlying PMOS makes it difficult for single-component interventions to comprehensively address its multiple pathological pathways; therefore, compound formulation strategies have become a key direction in the development of functional foods. In recent years, a research paradigm combining network pharmacology with experimental validation has yielded a series of advances in the field of PMOS, potentially providing a theoretical basis and translational pathways for the design of synergistic formulations derived from MFH.

**Figure 4:**
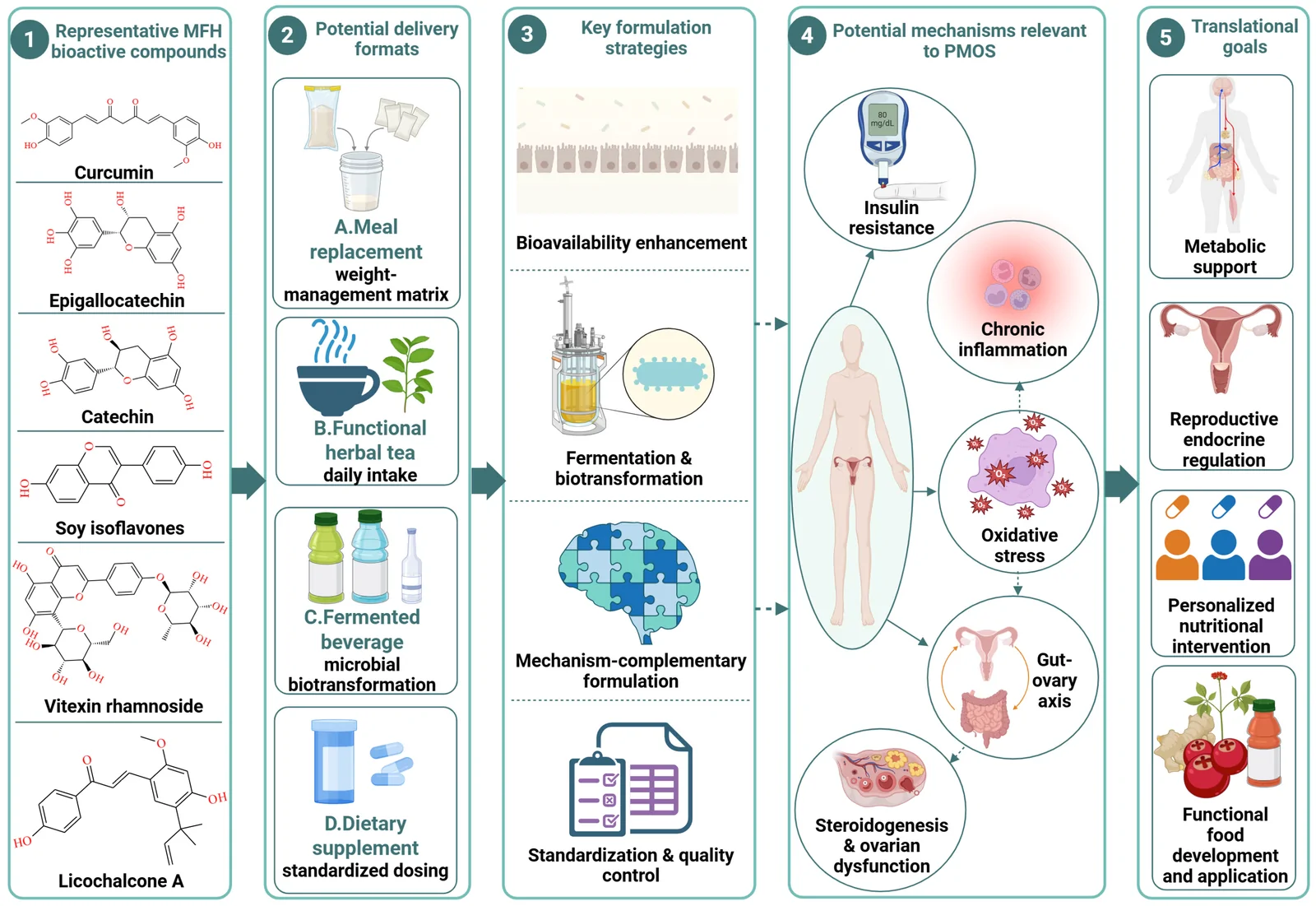
Integrated framework for MFH-derived functional food development targeting PMOS management. The schematic outlines a systematic strategy from compound selection and delivery format design to formulation optimization, mechanistic elucidation, and translational application. Key MFH bioactives (curcumin, catechins, cinnamon polyphenols, soy isoflavones, hawthorn flavonoids, and licorice flavonoids) are mapped to four delivery formats (meal replacement, herbal tea, fermented beverage, and dietary supplement) and linked to PMOS-relevant mechanisms (IR, steroidogenesis, OS, and the gut–ovary axis), with formulation strategies and translational goals providing a roadmap for evidence-based functional food development.

Choi et al. [138] used a combination of network pharmacology and a letrozole-induced PCOS rat model to demonstrate that 45 active components from the STH formula-including quercetin, shannaiol, and 45 other active components derived from MFH are enriched in the ovarian steroidogenesis and cell cycle pathways. These compounds can downregulate ovarian AMH and AMHR2 expression in a dose-dependent manner, reduce the number of cystic follicles, and restore corpus luteum and endometrial thickness, confirming that the MFH formula exerts synergistic therapeutic effects by regulating AMH-mediated ovarian dysfunction through multiple targets. In contrast, Wang et al. [139], using a DHEA-induced PCOS mouse model, revealed that quercetin—the core active ingredient in the “Wenkenshijiaotang” formula—acts by targeting IL-6, PTGS2, HIF1A, mTOR, and EGFR-key genes associated with OS and ferroptosis to inhibit oxidative damage and ferroptosis pathways in the ovaries, thereby improving ovarian morphology and sex hormone levels. This suggests that the therapeutic mechanisms of MFH formulations may exhibit differential preferences for specific pathological pathways depending on their composition. Furthermore, Hai et al. [140] found that three core components in Banxia Xiexin Decoction—quercetin, glycyrrhetinic acid A, and naringin—jointly target key OS-related genes such as FOS, JUN, MAPK3, TP53, and HSP90AA1, among other key genes associated with OS, and are enriched in the PI3K-AKT and MAPK signaling pathways. This indicates that this formula also targets OS as its primary intervention node; however, its multi-component synergistic action on a single pathway contrasts with the single-component-dominated mechanism of the Wenkenshijiaotang formula. The above studies indicate that although the MFH compound can synergistically intervene in various pathological stages of PMOS through multi-target mechanisms, its specific mechanisms vary depending on the composition of the compound. Based on this, compound design should adhere to two core principles: first, streamlining and standardization of components by conducting targeted enrichment and standardized extraction of active ingredients while preserving the original formula’s combination logic to ensure consistency across batches; second, mechanism complementarity, whereby MFH’s active ingredients, such as cinnamic acid polyphenols for improving IR, isoflavones for antioxidant effects, and curcumin for anti-inflammatory effects, act synergistically on different pathological pathways to achieve multi-target coverage and synergistic enhancement. These principles provide a practical translational pathway for adapting classical herbal formulas into functional foods. However, these design principles represent a translational framework rather than an evaluated product. Clinical validation of safety, efficacy, and batch consistency is required (Figure 4).

## 7. Current Challenges and Future Perspectives

### 7.1. Research-Level Bottlenecks

Current research on the active ingredient MFH in the management of PMOS remains primarily preclinical, with a severe shortage of high-quality RCTs. Existing clinical trials generally suffer from issues such as small sample sizes, short intervention periods, and inconsistent dosing; the certainty of evidence for most results in meta-analyses ranges from low to very low [141]. Elucidating the synergistic mechanisms of multi-component, multi-target formulations also faces methodological challenges; the traditional “single-component, single-target” paradigm struggles to explain the cross-regulation of dozens of components in PMOS steroid synthesis, insulin signaling, and inflammatory pathways [142]. Furthermore, the heterogeneity of PMOS diagnostic criteria and clinical endpoints results in poor comparability across studies and makes it difficult to establish dose–response relationships, thereby limiting the ability to demonstrate the efficacy of translating research evidence into product development [143].

### 7.2. Bottlenecks at the Translation Stage

The core obstacle at the translational level lies in the unclear dose–response relationship between the “effective dose” and the “food addition level.” In clinical studies, the intervention doses of active ingredients such as curcumin (500 mg per dose) are typically far higher than the amounts permitted by regulations for general food products, and the lack of PK-PD bridging studies means there is no scientific basis for determining reasonable addition levels [144]. Furthermore, most studies report only short-term intervention effects; there is a lack of systematic evaluation of the long-term stability of active ingredients in functional food matrices and the retention rate of bioactivity during the shelf life, which directly limits the commercial viability of these products [145]. The existence of these translational bottlenecks underscores the necessity of synergistically advancing both formulation optimization and regulatory science.

### 7.3. Future Directions

Future research should focus on the following three areas: (1) Organoid and organ-on-a-chip technologies: ovarian organoids derived from PMOS patients can reproduce key pathological features such as steroidogenesis dysregulation and arrested follicular development, providing a more physiologically relevant platform for high-throughput screening of active ingredients in MFH and personalized efficacy evaluation [146]; (2) AI-driven identification of active ingredients and formulation optimization: by integrating transcriptomics, network pharmacology, and machine learning, it is possible to systematically predict the multi-target regulatory networks of active ingredients in MFH, thereby transforming the formulation of compound preparations from an empirical approach to data-driven, intelligent optimization [147]; (3) International Multicenter Clinical Trials: using standardized PMOS diagnostic criteria and endpoint evaluation metrics, large-scale (>200 cases) and long-term (>6 months) randomized, double-blind, controlled trials will be conducted, incorporating subgroup analyses based on metabolic phenotypes to enhance the international recognition and level of evidence of the clinical findings [148].

### 7.4. Safety Considerations: Dosage, Toxicity, and Interactions

Dose–response relationships for MFH bioactives in functional foods remain poorly defined; clinical curcumin doses (e.g., 500–6000 mg/day) often exceed permitted food addition levels. Curcumin is generally well tolerated, with mainly mild gastrointestinal adverse events, but long-term toxicity data are limited. Licorice may cause hypertension and hypokalemia. Current safety data are predominantly derived from short-term studies of single constituents, with particular emphasis on curcumin, while evidence regarding MFH compound formulations, long-term daily consumption, and special populations (e.g., pregnant women, patients with hepatic or renal impairment, and those receiving concomitant medications) remains limited. Moreover, most studies have not systematically evaluated dose–response relationships, long-term toxicity, reproductive toxicity, or drug–drug interactions. Consequently, systematic long-term safety investigations are essential before MFH functional foods can be recommended for long-term PMOS management. Compound–compound interactions, such as curcumin–piperine, and food–drug interactions, including curcumin–warfarin (increased INR) and curcumin–antidiabetic drugs, require close monitoring. Systematic safety assessment is therefore essential before clinical translation. Consequently, prior to clinical translation, it is urgently necessary to conduct randomized controlled trials, comprehensive safety studies (including chronic toxicity and drug–drug interaction assessment), human bioavailability assessments accounting for food-matrix effects, and standardized product validation (encompassing formulation stability, batch-to-batch consistency, sensory acceptability, and scale-up feasibility).

## 8. Conclusions

This review systematically examines the mechanisms of action of bioactive compounds derived from MFH in the management of PMOS and the progress made in their development as functional foods. It clarifies their value in improving IR, regulating ovarian steroid synthesis, alleviating chronic inflammation and OS, restoring the gut microbiota–ovarian axis, and regulating the five levels of homeostasis in the hypothalamus–pituitary–ovarian axis. Current evidence suggests that active components derived from MFH, such as curcumin, quercetin, puerarin, soy isoflavones, and cinnamon polyphenols, can systematically intervene in multiple pathological pathways of PMOS by regulating core signaling pathways including PI3K/AKT, AMPK, NF-κB, and Nrf2/HO-1, thereby providing a preliminary molecular basis for their potential adjunctive treatment. Network meta-analyses further confirm that supplements such as omega-3, inositol, and curcumin offer evidence-based advantages in improving IR, lipid metabolism, and OS markers; however, the certainty of most clinical evidence remains low to moderate. Overall, the clinical evidence base remains limited by small sample sizes, short intervention durations, inconsistent dosing, and generally low or very low certainty. Therefore, the therapeutic effects of MFH bioactives in PMOS should be regarded as preliminary and require confirmation in adequately powered randomized controlled trials.

The complexity of the pathophysiological mechanisms underlying PMOS makes it difficult for single-component interventions to comprehensively address its multidimensional pathological network; in contrast, the MFH compound, through its “multi-target, multi-pathway” synergistic effects, offers a more promising translational pathway for the development of functional foods. The introduction of the 2025 national filing policy for compound health foods has provided a clear regulatory framework for the standardized development of MFH compound functional foods, while also opening a policy pathway for the adaptation of classical herbal formulas (such as Siwu Decoction) into functional foods.

However, the translation of research evidence into functional food products still faces multiple bottlenecks. At the research level, the lack of high-quality randomized controlled trials, difficulties in methodologically analyzing the synergistic mechanisms of multi-component formulations, and inconsistencies in clinical endpoints have hindered the elevation of the evidence level. At the translation level, the ambiguous dose–response relationship between “effective dose” and “food addition level,” as well as the lack of data verifying long-term stability and shelf life, directly limit the commercial viability of these products. To overcome these bottlenecks and achieve a transition for MFH functional foods from “empirical application” to “precision interventions guided by evidence-based medicine,” there is an urgent need to foster synergistic collaboration across three dimensions: research paradigms, technological development, and industrial translation. At the research paradigm level, patient-derived ovarian organoids and organ-on-a-chip technologies from PMOS patients can provide a more physiologically relevant platform for active ingredient screening, while AI-driven multi-omics integration and network pharmacology analysis hold promise for the intelligent design and optimization of compound formulations. At the technological development level, the integrated application of advanced technologies, such as ultrasound-assisted extraction, nanoencapsulation, and targeted controlled-release systems, offers viable technical pathways to overcome bottlenecks in bioavailability and stability challenges associated with active ingredients. In this context, the development of food-grade delivery systems should be prioritized. Compared with pharmaceutical nanocarriers, lipid-based systems, biodegradable polymer nanoparticles prepared with food-grade or GRAS-certified materials, and stabilization technologies already applied in the food industry are more suitable for functional food applications. Future studies should systematically evaluate their stability in food matrices, sensory acceptability, and scale-up feasibility to facilitate the translation of MFH bioactives from mechanistic research to functional food products. These efforts should be aligned with industrial translation to ensure that food-grade delivery systems can be manufactured at scale. At the industrial translation level, there is an urgent need to conduct large-scale, long-term, multicenter international randomized controlled trials using standardized PMOS diagnostic criteria and endpoint evaluation metrics to accumulate high-level evidence-based data. Beyond efficacy and formulation, safety research represents another critical dimension for clinical translation, encompassing dose–response characterization, long-term toxicity assessment, compound–compound interactions, and food–drug interaction studies. These safety considerations should be integrated into the proposed research paradigms, technological development, and industrial translation efforts to ensure the safe translation of MFH functional foods.

In summary, MFH bioactive compounds may hold potential therapeutic promise and application prospects in the comprehensive management of PMOS. With advances in basic research, formulation technology, and regulatory policy, MFH functional foods may gradually move from an adjunctive role toward a more integrated position in PMOS management, potentially offering nutritional strategies that complement existing interventions. However, these possibilities should be interpreted with caution, as current clinical evidence remains limited and further randomized controlled trials are needed. This review also aims to provide a systematic theoretical framework and practical guidance across the research continuum, encompassing active ingredient discovery, mechanistic elucidation, formulation optimization, and product commercialization.

## Data Availability

No new data were created or analyzed in this study. Data sharing is not applicable to this article.
